# Exposure to high altitude leads to disturbances in host metabolic homeostasis: study of the effects of hypoxia-reoxygenation and the associations between the microbiome and metabolome

**DOI:** 10.1128/msystems.01347-24

**Published:** 2025-04-16

**Authors:** Qin Zhao, Doudou Hao, Siyu Wang, Siyuan Chen, Chaohua Zhou, Chen Fan, Qian Su, Wenting Huang, Jiaxin Liu, Qingquan Kong, Yunhong Wu, Zeng He

**Affiliations:** 1 Department of Biobank, Hospital of Chengdu Office of People’s Government of Xizang Autonomous Region (Hospital.C.X.) 569168 , Chengdu, Sichuan, China; 2 Department of Science Education, Hospital of Chengdu Office of People’s Government of Xizang Autonomous Region (Hospital.C.X.) 569168 , Chengdu, Sichuan, China; 3 State Key Laboratory of Genetic Resources and Evolution/Key Laboratory of Healthy Aging Research of Yunnan Province, Kunming Institute of Zoology, Chinese Academy of Sciences 53028 , Kunming, Yunnan, China; 4 Stomatology, Hospital of Chengdu Office of People’s Government of Xizang Autonomous Region (Hospital.C.X.) 615807 , Chengdu, Sichuan, China; 5 Institute of Blood Transfusion, Chinese Academy of Medical Sciences and Peking Union Medical College 70567, Chengdu, Sichuan, China; 6 Department of Orthopedics, Hospital of Chengdu Office of People’s Government of Xizang Autonomous Region (Hospital.C.X.) 615801 , Chengdu, Sichuan, China; 7 Department of Endocrinology and Metabolism, Hospital of Chengdu Office of People’s Government of Xizang Autonomous Region (Hospital.C.X.) 615797 , Chengdu, Sichuan, China; North Carolina Agricultural and Technical State University, Greensboro, North Carolina, USA

**Keywords:** hypoxia, reoxygenation, plateau population, gut microbiota, metabolism

## Abstract

This study investigated alterations in hematological parameters, gut microbiota composition, and fecal and plasma metabolic profiles among high-altitude residents during reoxygenation periods of 1 week, 1 month, and 4 months to elucidate the effects of reoxygenation on human physiology and metabolism. Exposure to high altitudes alters intestinal flora, plasma and fecal metabolites, disrupting their metabolic balance. Distinct differences in amino acid, lipid, energy, immune, cofactor, and vitamin metabolism pathways were detected between high- and low-altitude populations, with a partial recovery of disparities during reoxygenation. Although the gut microbiota exhibited limited adaptive homeostasis to altitude variations, the abundance of microbial taxa and the expression levels of fecal metabolites during the initial reoxygenation phase, particularly during the first week, were sensitive to the reoxygenated environment. Through 16S rRNA gene sequencing and bioinformatics analysis, operational taxonomic units (OTUs) were annotated at the genus level, revealing that the genera *Barnesiella, Parabacteroides, and Megasphaera*, along with plasma L-arginine, S1P, and alpha-D-glucose, emerged as potential biomarkers for the first week of reoxygenation among high-altitude populations. Notably, a marked change in oxidative stress levels and an increase in antioxidant capacity were observed in high-altitude residents during early reoxygenation. Tyrosine metabolism, which is jointly regulated by the plasma and fecal metabolites and gut microbiota, plays an important role under high-altitude conditions during initial reoxygenation. Additionally, the plasma metabolites pyridoxine and hypoxanthine and the *Rothia* genus correlated significantly with high-altitude deacclimatization syndrome scores during the first week of reoxygenation.

**IMPORTANCE:**

Our research focuses on the prompt activation of tyrosine metabolism in plasma following reoxygenation, along with the regulatory mechanisms employed by the intestinal microbiota and the metabolism of feces to modulate this metabolic process. Notably, in the initial stages of reoxygenation, specific microbial genera such as *Barnesiella, Parabacteroides*, and *Megasphaera*, alongside plasma biomarkers including L-arginine, S1P, and alpha-D-glucose, emerge as pivotal players. Additionally, our findings reveal a distinct hematological profile characterized by a decrease in the MCHC and increases in the MCV and RDW-SD during the first week of reoxygenation, and this temporal window marked a crucial juncture in the plasma metabolome. Whereas the first month of reoxygenation signified a pivotal phase in the gut microbiome’s adaptation to altered environmental conditions, as evidenced by alterations in alpha diversity.

## INTRODUCTION

The Qinghai‒Tibet Plateau, often referred to as the “roof of the world,” is inhabited by approximately 140 million individuals (1). Globally, more than 35 million individuals travel, participate in sports, or engage in work activities in high-altitude regions annually (2). Owing to the frequent migration of individuals between plateaus and plains, the phenomenon of deacclimatization from high altitudes has garnered significant attention as a pressing health concern with profound implications for broader scientific research and public health policies.

Extended exposure to high altitudes can lead to multiple physiological dysfunctions, including cerebral microcirculatory disorders (3), spatial memory impairment (4), reduced bone turnover, elevated blood viscosity, lung injury, and sympathetic nervous system excitation. Numerous studies have reported significant pathological changes in the brain, liver, kidney (5), and heart (6) of rats exposed to acute hypoxia followed by reoxygenation. Additionally, abnormal electroencephalogram (EEG) patterns in individuals residing at high altitudes for more than a year are positively correlated with altitude (7). Short-term acute exposure to high altitude can lead to significant executive function and memory deficits among healthy children, but these effects typically resolve within 3 months of returning to low altitude (8). Patients with high-altitude deacclimatization syndrome may require up to 100 days to fully recover to their previous physiological state (9). Although these symptoms often improve upon relocating to lower altitudes, physiological parameters require a considerable amount of time to fully revert to normal levels.

Numerous studies have demonstrated distinct gut microbiomes and metabolic profiles in individuals upon their return to a low-altitude environment that differ from those observed in long-term residents in high-altitude environments and at low altitude. Altitude significantly influences the composition and relative abundance of the gut microbiota, with distinct bacterial genera prevailing at varying altitudes (10). The abundant microbes in the intestines of Tibetan natives are strongly associated with carbohydrate metabolism, and their fecal microbiota displays a greater diversity than that of Han Chinese immigrants (11). Moreover, individuals who resided at 5,300 m and 5,170 m for 12 months and subsequently returned to plain for 6 months presented perturbations in phenylalanine metabolism and elevated levels of the pentose phosphate pathway (12). The evidence suggests that the gut microbiota plays a pivotal role in modulating the plasma metabolome (13). Consequently, we postulated that substantial alterations in the gut microbiota and metabolism occurred during the transition from hypoxia to reoxygenation as individuals migrate from the plateau to the plains.

Given the substantial variations in oxygen concentration, climate, and available food sources between high and low altitudes, these environmental factors could exert profound effects on individuals who frequently travel between the two environments. The aim of this study was to comprehensively examine the changes in the gut microbiota, fecal metabolism, and plasma metabolism among residents who have lived on the plateau for a long period and then migrated to the plains and to elucidate the interaction mechanisms between the gut microbiota and metabolism. This comprehensive exploration aimed to offer novel insights into the impact of environmental shifts on human health. In an era of increasing globalization and interregional mobility, the lessons learned from these remarkable environments have profound implications for promoting healthy living and sustainable development across diverse landscapes.

## MATERIALS AND METHODS

### Recruited volunteer information

**Fig 1:**
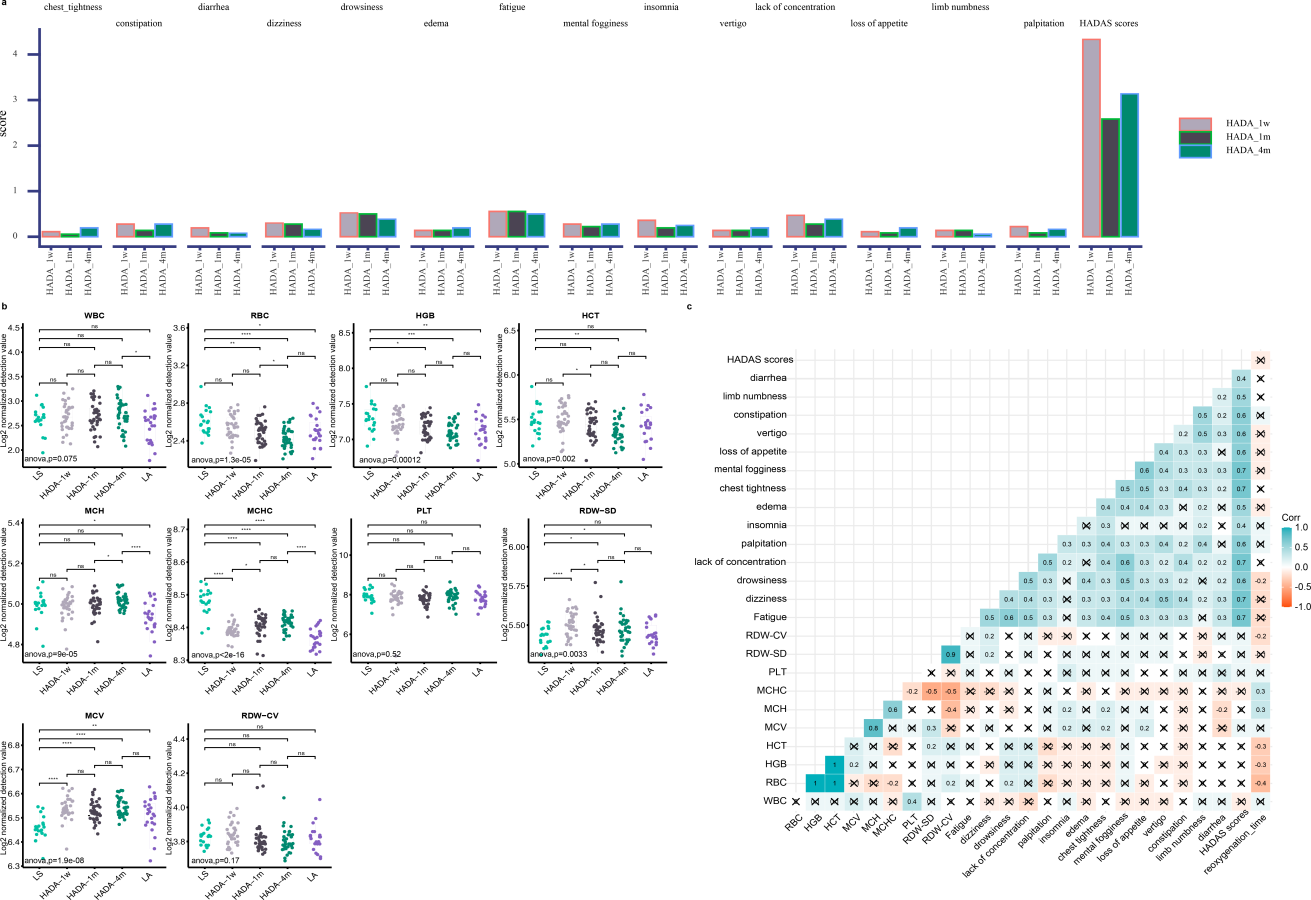
Comprehensive assessment of physiological adaptation and blood parameters among high-altitude residents upon transition to low altitude: HADAS scores, questionnaire indicators, and blood routine analysis. (**a**) Average high-altitude deacclimatization syndrome (HADAS) scores and changes in questionnaire indicators among high-altitude residents after arrival at the plains. (**b**) Differential analysis of blood routine parameters (white blood cell count (WBC), red blood cell count (RBC) , hemoglobin (HGB), hematocrit (HCT), mean corpuscular hemoglobin (MCH), mean corpuscular hemoglobin concentration (MCHC), platelet count (PLT), red blood cell distribution width-standard deviation (RDW-SD), mean corpuscular volume (MCV), red blood cell distribution width-coefficient of variation (RDW-CV) during hypoxia and reoxygenation among high-altitude residents. The difference between the two groups was investigated using *t*-test, whereas ANOVA was employed to assess the overall significance of differences across groups. (**c**) Spearman correlation analysis between blood routine parameters, acclimatization loss scores, and acclimatization loss duration during the reoxygenation period (HADA-1w, HADA-1m, and HADA-4m groups) among high-altitude residents, assessed through Spearman correlation analysis.

This study included 59 healthy volunteers aged between 20 and 50 years. For the high-altitude population, 21 volunteers were recruited from Lhasa, and an additional 15 high-altitude volunteers joined us within 7 days after traveling from Lhasa to Chengdu. There is no significant difference between the high-altitude population recruited from the plateau and that recruited from the plain (Fig. S1). During the reoxygenation phase in Chengdu, participants from high-altitude populations were administered the high-altitude deacclimatization syndrome (HADAS) questionnaire to assess their symptoms. Twenty-three low-altitude (LA) volunteers were recruited from Chengdu. Volunteers were excluded if they met any of the following criteria: (i) had an altitude acclimatization duration of less than 1 year; (ii) had a history of infection with any of four blood-borne infectious diseases (hepatitis B, hepatitis C, HIV, or *Treponema pallidum* (TP); (iii) had an age outside the specified range (i.e., <18 or >60 years); (iv) had a history of anemia or a hemoglobin concentration not within the normal range for the respective group (normal group: 130 g/L ≤ male ≤ 175 g/L; 115 g/L ≤ female ≤ 150 g/L; high-altitude polycythemia group: male ≥210 g/L; women ≥ 190 g/L); (v) had chronic diseases affecting metabolism, such as diabetes, hypertension, or cancer; or (vi) had any other conditions deemed unsuitable for inclusion. Blood and fecal samples were systematically procured from participants residing at high altitudes, encompassing time points 1 week prior to their relocation to Chengdu (LS), as well as within the first week (high-altitude deacclimatization, HADA_1 w), 1 month (HADA_1 m), and 4 months (HADA_4 m) following their arrival in Chengdu. A questionnaire was administered at each time point. Fecal and blood samples were collected directly from the 23 low-altitude volunteers. The precise sampling information is outlined in Fig. 1; Table 1. After collection, 300 µL of each blood sample was reserved for routine blood testing, and the remaining blood was centrifuged at 3,000 rpm for 10 min. Subsequently, 300 µL of the isolated plasma was transferred to a cryovial and stored at −80°C. All the samples were stored in the Biobank of the Hospital of Chengdu Office of People's Government of Xizang Autonomous Region. Blood samples exhibiting hemolysis were excluded from the study. Upon collection, the fecal samples were stored in cryovials and immediately snap-frozen in liquid nitrogen. Samples that fell short of the required quantity due to difficulties in producing sufficient feces at the time of collection or due to misinterpretation of the instructions given to participants concerning the minimum amount needed were excluded. Additionally, samples obtained from individuals who had recently consumed antibiotics were also excluded from the study.

**TABLE 1:** Summary of demographic and usage characteristics of the sample

Characteristics		Effective sample number		Age (mean ± SD)	Gender		Plateau residence time (years)		
		Plasma sample	Stool sample		Female	Male	0	3–5	20–30
CD (*N* = 23)	LA	22	21	28.83 ± 4.65	15 (25.42%)	8 (13.56%)	23 (38.98%)	0 (0%)	0 (0%)
HA (*N* = 36)	LS	21	16	22.78 ± 1.15	20 (33.90%)	16 (27.12%)	0 (0%)	9 (15.25%)	27 (45.76%)
	HADA_1 w	35	32						
	HADA_1 m	35	32						
	HADA_4 m	36	36						
Total (*N* = 59)	/^*a*^	149	137	25.14 ± 4.22	35 (59.32%)	24 (40.68%)	23 (38.98%)	9 (15.25%)	27 (45.76%)

^*a*^ “/” represents not applicable.

### Multiomics approach

#### Intestinal flora 16S rRNA sequencing

After thawing on ice, the fecal samples were thoroughly mixed and centrifuged, followed by DNA extraction. DNA quality was assessed using a Qubit fluorometer and an enzyme labeling instrument. Thirty nanograms of genomic DNA of satisfactory quality, along with the corresponding fusion primers (F：ACTCCTACGGGAGGCAGCAG, R：GGACTACHVGGGTWTCTAAT), were used to prepare a PCR mixture for amplification of the 16S rRNA V3-V4 region. The amplified products were purified with Agencourt AMPure XP beads, eluted in the elution buffer, and labeled for complete library preparation. The fragment size and concentration of the library were subsequently analyzed using an Agilent 2100 Bioanalyzer. The qualified library was then sequenced on the Illumina HiSeq and MGISEQ-2000 platforms (BGI, China) on the basis of the size of the inserted fragments. The sequencing data were processed with Cutadapt v2.6 to obtain clean data. The paired-end sequencing reads were assembled into sequences using FLASH software (v1.2.11), yielding tags from the hypervariable regions. Finally, the tags were clustered into operational taxonomic units (OTUs) on the basis of 97% sequence similarity using USEARCH software (v7.0.1090_i86linux32). The strengthening the organization and reporting of microbiome studies (STORMS) checklist can be found at GitHub (https://github.com/langlibaitiaoshuafeidao/Exposure-to-high-altitude-leads-to-disturbance-of-host-metabolic-homeostasis/blob/09c4eda6e46f4db02a937f057eda293e28dcef72/Supplementary%20Information/STORMS_Excel_1.03.xlsx).

#### Intestinal flora bioinformatics analysis

Using R (v3.4.1), species classification of OTUs was conducted through Ribosomal Database Project (RDP) database comparison, and bar graphs of microbiota abundance were generated for each sample at the phylum, class, order, family, genus, and species levels. OTUs with species abundances less than 0.5% or those without a specific species annotation were grouped into a category labeled “Others” to ensure a comprehensive representation of the microbial community. Following data preprocessing, clustering, and annotation, the OTUs were counted for each individual sample. The abundance of each taxonomic category (phylum, class, order, family, genus, and species) was calculated by summing the abundances of OTUs that belonged to the same taxonomic category. Additionally, Kruskal‒Wallis tests were conducted to identify species with significant differences (*P* < 0.05) among the groups. The alpha diversity was quantified with the *t* test, facilitated by the MicrobiotaProcess and phyloseq packages. For beta-diversity analysis, the vegan package was used to calculate Bray‒Curtis distances, which were then subjected to principal coordinate analysis (PCoA). Additionally, the reshape2 package was used to conduct boxplot-based differential analysis based on the Bray‒Curtis distances. Linear discriminant analysis effect size (LEfSe) was conducted with LEfSe (https://huttenhower.sph.harvard.edu/galaxy/). Moreover, PICRUSt2 (v2.2.0-b) was used to functionally annotate the bacterial communities via the Kyoto Encyclopedia of Genes and Genomes (KEGG) database. The specific functional genes were assigned KEGG orthology (KO) identifiers, and metabolic pathway information was retrieved from the KEGG database (Table S1). Differences in functionality were identified with the Wilcoxon test (for two groups).

#### Metabolomic analyses of stool and plasma samples

To prepare fecal samples for metabolomic analysis, 25 mg of thawed feces was mixed with precooled extraction solution (methanol:acetonitrile:water = 2:2:1, vol:vol:vol). The mixture was ground at 50 Hz for 5 min and then sonicated in a water bath at 4°C for 10 min. Following incubation at −20°C for 1 h, the sample was centrifuged at 25,000 × *g* for 15 min at 4°C. The supernatant was then dried using a vacuum concentrator and resuspended in reconstitution solution (methanol:water = 1:9, vol:vol). The resuspended sample was vortexed for 1 min, sonicated in a water bath at 4°C for 10 min, and centrifuged again at 25,000 × *g* for 15 min at 4°C. The supernatant was collected for metabolomic analysis.

For the plasma samples, 100 µL of plasma was mixed with extraction solvent containing internal standard 1 (methanol:acetonitrile:water = 4:2:1, vol/vol/vol). The mixture was vortexed for 1 min, incubated at −20°C for 2 h, and then centrifuged at 25,000 × *g* for 15 min at 4°C. A total of 600 µL of the supernatant was transferred and concentrated with a vacuum concentrator. The residue was resuspended in reconstitution solution (methanol:pure water = 1:1, vol/vol) and vortexed for 10 min until it was dissolved. The sample was then centrifuged at 25,000 × *g* for 15 min at 4°C, after which the supernatant was collected for metabolomic analysis.

#### Ultra-performance liquid chromatography–mass spectrometry (UPLC‒MS)

In this study, a Waters 2777C UPLC (Waters, USA) instrument coupled with a Q Exactive HF high-resolution mass spectrometer (Thermo Fisher Scientific, USA) was used for the separation and detection of metabolites. Chromatographic separations were performed using a BEH C18 column (1.7 µm, 2.1 × 100 mm; Waters, USA). The mobile phase consisted of 0.1% formic acid in water (solvent A) and 0.1% formic acid in methanol (solvent B) for positive ion mode, whereas 10 mM ammonium formate in water (solvent A) and 10 mM ammonium formate in 95% methanol (solvent B) were used for negative ion mode. The gradient elution conditions were as follows: 0–1 min, 2% B; 1–9 min, 2%–98% B; 9–12 min, 98% B; 12–12.1 min, 98%–2% B; and 12.1–15 min, 2% B. The flow rate was maintained at 0.35 mL/min, and the column temperature was set to 45°C. A sample injection volume of 5 µL was used. For mass spectrometry, the Q Exactive HF mass spectrometer was operated in both full scan and data-dependent MS/MS modes. The scan range was set from 70 to 1050 m/z, with resolutions of 120,000 for full scan and 30,000 for MS/MS. The automatic gain control (AGC) targets were set to 3e6 for full scan and 1e5 for MS/MS, with maximum injection times of 100 ms and 50 ms, respectively. The fragmentation energy levels were set to 20, 40, and 60 eV. The ESI source parameters were optimized, with a sheath gas flow rate of 40, an auxiliary gas flow rate of 10, a spray voltage of 3.80 kV for positive ion mode and 3.20 kV for negative ion mode, a capillary temperature of 320°C, and an auxiliary gas heater temperature of 350°C. The raw MS data were imported into Compound Discoverer 3.3 software (Thermo Fisher Scientific, USA, v.3.3) for analysis, which was integrated with the BGI metabolome database (BMDB) and the mzCloud and ChemSpider online databases for metabolite identification.

#### Bioinformatic analysis of metabolites

The original MS data were subjected to metabolite peak extraction and identification to derive crucial information such as molecular weight, retention time, peak area, and identification of the metabolites. The software export results were subsequently subjected to further preprocessing with metaX, which yielded compounds and quantitative values suitable for formal analysis. The metabolites were annotated through databases such as the KEGG database and the human metabolome database (HMDB), providing details such as the KEGG ID, HMDB ID, category, and metabolic pathways in which they participate within the KEGG framework. It is worth noting that not all metabolites can be comprehensively annotated and defined. Additionally, the mean and standard deviation (SD) of each metabolite across different sample groups were computed, enabling the calculation of fold changes (FC) among the comparison groups. Statistical significance testing of metabolite expression differences across comparison groups was performed with Student’s *t* test, which yielded *P* values. These *P* values were then adjusted with the Benjamini–Hochberg algorithm to obtain q values. The metabolome results were analyzed with the BGI Dr. Tom platform (https://biosys.bgi.com, accessed on March 19, 2024).

To analyze the overall difference between the two groups, we used partial least squares discriminant analysis (PLSDA) and orthogonal partial least discriminant analysis (OPLS-DA). Differentially abundant metabolites were subsequently identified with the variable importance in projection (VIP) values obtained from the OPLS-DA, alongside the FC and *P* values obtained from the univariate analysis. The screening criteria for differentially expressed metabolites (DEMs) in this study were as follows: OPLS-DA VIP value ≥1, FC ≥ 1.2 or ≤0.83, and *P* value < 0.05 (Tables S2 and S3). Pathway enrichment analysis of these metabolites was conducted with the KEGG database (Table S4). The differential abundance (DA) score assesses the overall alteration in the abundance of all metabolites within a specific pathway. The temporal trends in gene expression were analyzed, and clusters were identified with the Mfuzz package in R. Functional enrichment analysis of metabolites within each cluster was performed with the clusterProfiler package in R. Multiomics correlation analyses using Spearman methods were performed with the psych package in R. The resulting intestinal flora-metabolite-pathway network was visualized with Cytoscape software. Heatmaps were generated for metabolite analysis with the complexheatmap package in R software. Distance-based redundancy analysis (dbRDA) was performed with the vegan and rdacca.hp packages in R. Mantel correlation analysis was conducted in the Hy4m/linkET package with R. Random forest analysis was performed with the randomForest package in R. A random forest regression model was used utilizing 10-fold cross-validation. The entire data set was divided into a training set (comprised of 70% of the data set) and a testing set (comprised of 30% of the data set). The metric chosen to assess the importance of predictive variables was an increase in node purity (IncNodePurity). Differences in hematological parameters, metabolite expression levels, and relative abundances of bacterial genera between groups were statistically analyzed with ANOVA, with t tests conducted where appropriate, utilizing the ggpubr R package. Graphical outputs were generated with R version 4.3.1.

## RESULTS

### Clinical characteristics of the hypoxia/reoxygenation period

During reoxygenation, the HADAS scores peaked in the first week for high-altitude inhabitants, with the highest averages observed for fatigue and drowsiness (Fig. 1a). Significant differences were noted in the baseline levels of RBC, HGB, MCH, MCHC, and MCV values between high-altitude residents and those residing at low altitudes (Fig. 1b). Upon arrival at Chengdu from Lhasa, high-altitude inhabitants experienced a significant decrease in MCHC and increases in MCV and RDW-SD during the first week. After 4 months of reoxygenation, all hematological indices, except for the WBC, MCH, and MCHC, which remained significantly greater than those of low-altitude residents, did not differ significantly. To further investigate the correlations between HADAS scores, reoxygenation duration, and hematological parameters, we conducted a correlation analysis. No significant correlation was found between hematological indices and deacclimatization scores. However, weak correlations were observed between reoxygenation time and drowsiness (one of the deacclimatization symptoms), RDW-CV, HCT, HGB, RBC, MCHC, and MCH (Fig. 1c). DbRDA was utilized to assess the associations among routine blood indices, the intestinal microbiota, and metabolites. Previous studies have demonstrated that hypoxia stimulates RBC production, leading to elevated levels of HGB and HCT. In the present investigation, the dbRDA model explained 70.09% of the variance in the plasma metabolome variance observed along axis 1, identifying MCHC, RDW_CV, and HGB as key environmental variables. These variables significantly modulated the gut microbiota, fecal metabolome, and plasma metabolome profiles during hypoxia‒reoxygenation transitions (Fig. S2).

### Characteristics of the intestinal flora during the hypoxia–reoxygenation period

**Fig 2:**
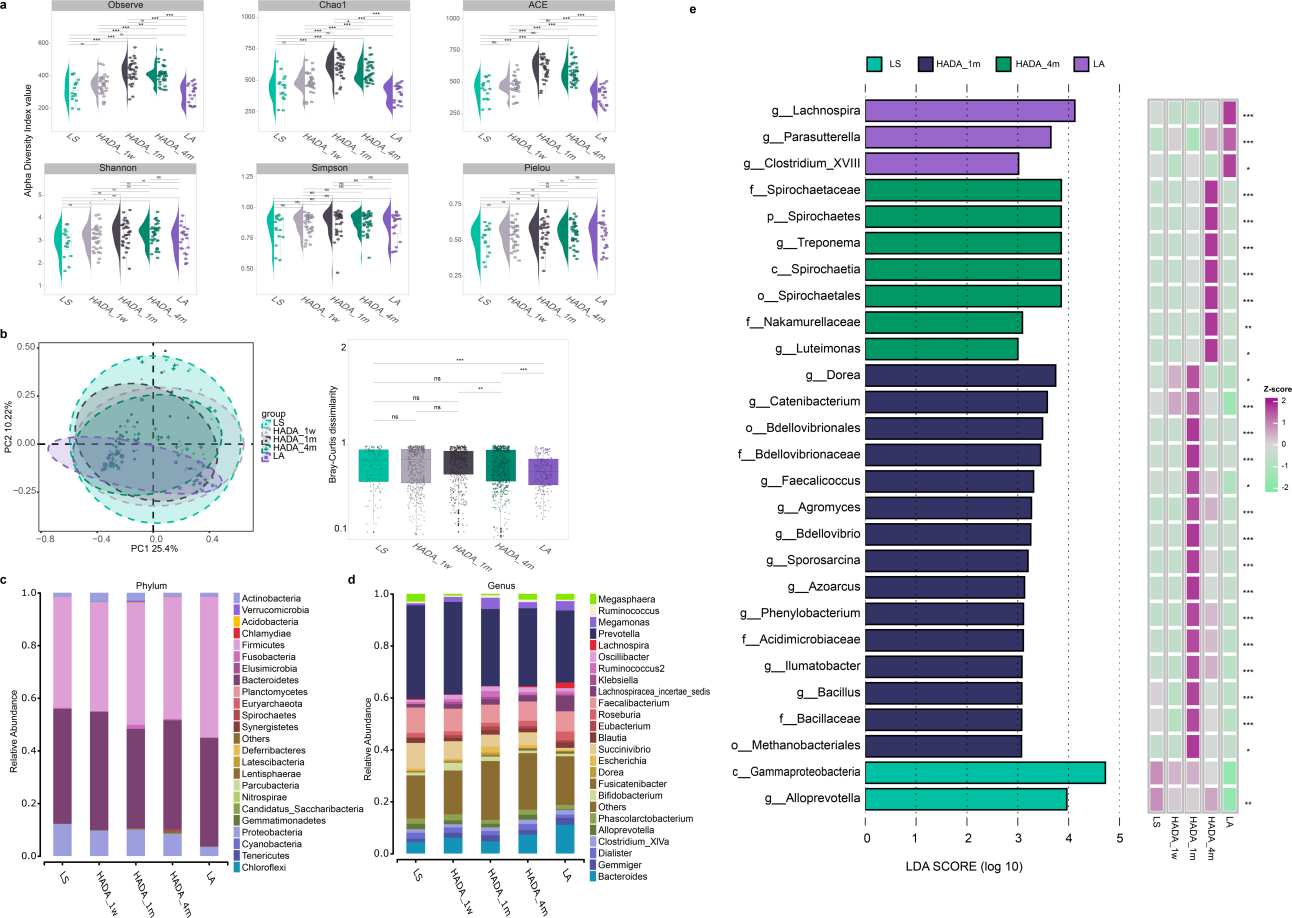
Comprehensive analysis of gut microbiota diversity and biomarkers among different groups. (**a**) Alpha diversity analysis of intestinal microbiota. (**b**) PCoA analysis of beta-diversity of gut microbiota. Boxplots of significance of beta-diversity (calculated as Bray-Curtis dissimilarity) differences between different groups were determined using wilcox.test analysis. *X*-axis labels correspond to the individual groups. (**c, d**) Histogram at the phylum and genus levels of intestinal microbiota. Species whose group average abundance is less than 0.5%, and all species not annotated at this taxonomic level are combined into Others. (e) LEfSe analysis screens the biomarkers based on *P* < 0.05 and LDA scores > 3. The heatmap displays the relative abundance differences of molecular biomarkers among different groups. The Kruskal-Wallis single-factor test was used to compare the relative abundance expression differences among multiple groups (*P < 0.05, **P < 0.01, ***P < 0.001).

To determine the potential correlation between the gut microbiota and hypoxia‒reoxygenation in high-altitude populations, we performed 16S rRNA gene sequencing on five groups of fecal samples. Our analysis revealed that the alpha diversity of the gut microbiota, as indicated by the Observed, Chao1, ACE, and Shannon indices, peaked at 1 month of reoxygenation (Fig. 2a). Additionally, PCoA and boxplot analyses demonstrated significant differences in beta-diversity between the LS and LA groups, as well as during the transition from one to 4 months of reoxygenation. Notably, significant differences in beta diversity were also observed between high-altitude and low-altitude individuals after 4 months of reoxygenation (Fig. 2b). Bacteroidetes and Firmicutes emerged as the dominant bacterial phyla in the present study. Specifically, the abundance of Bacteroidetes exhibited a dynamic pattern after reoxygenation, increasing from 43.63% to 45.05% within the first week, declining to 37.76% after 1 month and subsequently increasing to 41.46% at 4 months. Notably, the abundance of Bacteroidetes at 4 months after reoxygenation surpassed that observed at Lhasa and approximated the level (47.18%) found in individuals residing at LA. Conversely, the abundance of Firmicutes progressively increased after reoxygenation, with relative abundances of 41.56%, 46.30%, and 46.34% at HADA_1 w, HADA_1 m, and HADA_4 m, respectively. Although these abundances surpassed those recorded at Lhasa (42.18%), they remained below those observed in LA populations (53.58%) (Fig. 2c). The Firmicutes/Bacteroidetes (F/B) ratio fluctuated after reoxygenation, initially decreasing, then increasing, and finally decreasing again, although these changes were not statistically significant (*P* > 0.05). *Prevotella*, the predominant bacterial genus among high-altitude inhabitants (14), displayed a decreasing trend in abundance from 36.02% at high altitude to 35.57% after 1 week of reoxygenation, with a further decline to 29.68% at 1 month, followed by stabilization at 30.02% at 4 months (HADA_4 m) (Fig. 2d). Nevertheless, even at this stable level, the abundance of *Prevotella* remained greater than that in LA populations (27.75%) (Fig. 2d). LEfSe was employed to identify key bacterial taxa in each group (*P* < 0.05, LDA > 3) (Fig. 2e). Elevated abundances of *Alloprevotella* in the LS group correlated with disorders of the intestinal, hepatic, urinary, ocular, neurological, and cardiovascular systems. Conversely, *Agromyces, Dorea, Bacillus, Faecalicoccus, Bdellovibrio, Phenylobacterium, Sporosarcina, Catenibacterium, Azoarcus*, and *Ilumatobacter* presented increased abundances in the HADA_1 m group. *Luteimonas* and *Treponema* were enriched in the HADA_4 m group. *Parasutterella*, *Clostridium_XVIII*, and *Lachnospira* were enriched in the LA group.

To further elucidate the adaptation process of gut microbial communities to the plains environment, we conducted a multigroup differential abundance analysis on key bacterial taxa identified through LEfSe analysis (Fig. S3a). Notable differences were observed in the abundances of genera such as *Alloprevotella* and *Catenibacterium* between the high-altitude and plain populations. Moreover, *Dorea* exhibited varying abundances across different stages (1–4 months) after migration to the plains. Notably, genera including *Faecalicoccus, Agromyces, Sporosarcina, Azoarcus, Phenylobacterium, llumatobacter, Bacillus, and Luteimonas* exhibited particularly prominent changes in abundance during the initial migration period (1 week to 1 month). The discrepancy in the abundance of the *Treponema* genus between the initial migration phase and high-altitude populations suggests a potential stress-related response. Furthermore, the long-term alterations in *Lachnospira* and its differential abundance compared with that of the plains populations underscore the profound impact of migration on the gut microbiota. Finally, the differences in the abundances of the *Parasutterella* and *Clostridium_XVIII* genera confirmed the distinct gut microbial compositions between the high-altitude and plain populations. Furthermore, the relative abundances of *Agromyces*, *Alloprevotella*, *Phenylobacterium*, and *Treponema* were significantly correlated with reoxygenation duration (Fig. S3b). The prediction of KEGG pathways with PICRUSt based on 16S rRNA data revealed differences in microbial metabolic pathways, particularly in xenobiotic biodegradation and metabolism, amino acid metabolism, carbohydrate metabolism, and metabolism of cofactors and vitamins, across groups (Fig. S3c). Collectively, our findings suggest that the gut microbiota plays a pivotal role in the hypoxia-reoxygenation process experienced by high-altitude populations. By dynamically adjusting its diversity and composition, the gut microbiota adapts to environmental changes, thereby influencing the host’s health status and physiological functions.

### Characteristics of the fecal metabolome during the hypoxia–reoxygenation period

**Fig 3:**
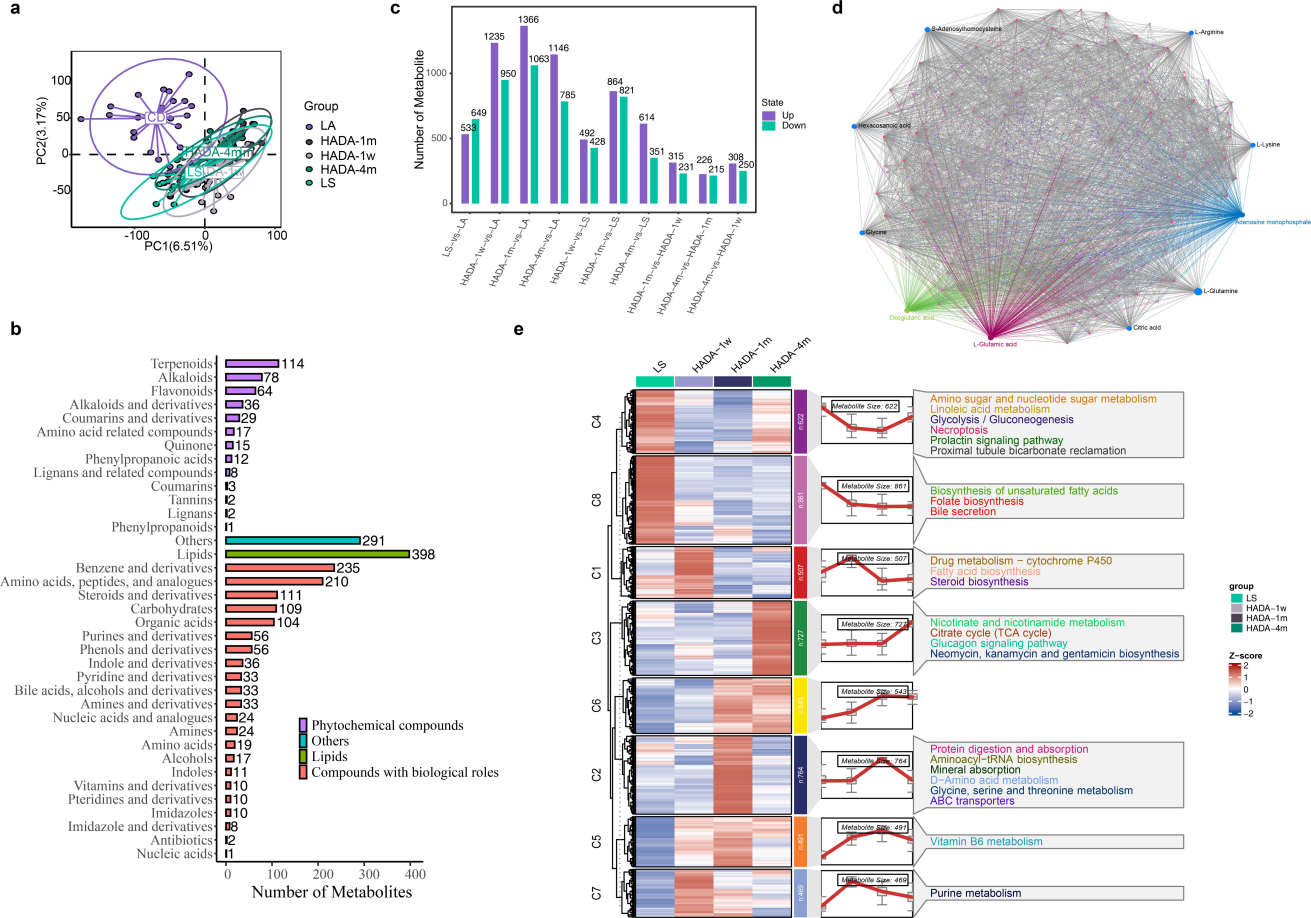
Overall characteristics of the fecal metabolome. (**a**) PLSDA analysis of fecal metabolome in each group. (**b**) Classification of detected fecal metabolite types, with counts based on the final class. Metabolites belonging to the lipid category are combined and displayed together. Metabolites not belonging to the categories of compounds with biological roles, phytochemical compounds, or lipids are represented as “Others” (**c**) Presentation of the number of differential metabolites in each comparison group. (**d**) Network analysis of total metabolites on the MetaboAnalyst website, where the size of the nodes represents the degree of correlation for each metabolite. The top 10 metabolites with the most relationship pairs are labeled with their names. Purple represents the network of L-glutamic acid with other metabolites, blue lines represent the network of adenosine monophosphate with other metabolites, and green lines represent the network of oxoglutaric acid with other metabolites. (**e**) Mfuzz analysis of temporal trends in metabolites and clustering, followed by KEGG pathway enrichment for each cluster’s metabolites. Only significantly enriched KEGG pathways (*P* < 0.05) are shown in the figure.

The PLSDA effectively segregated the LA group from the low-altitude residents in the LS group (Fig. 3a). However, during reoxygenation, the fecal metabolomic profiles of the plateau population were difficult to distinguish. Upon classifying the identified metabolites based on their final class, it was observed that lipids comprised the most significant group (Fig. 3b). DEMs were identified across various groups, resulting in a total of 4,985 DEMs. An examination of the differentially abundant metabolite counts revealed that the maximum number of differentially expressed metabolites (1,682 DEMs) between HADA and LS occurred during the HADA_1 m period of reoxygenation, with a subsequent decrease at HADA_4 m (992 DEMs) (Fig. 3c). Similarly, compared with the LA group, HADA_1 m presented the greatest number (2,429 DEMs) of differentially abundant metabolites (Fig. 3c). These findings were in line with the gut microbiota results, indicating that the greatest divergence in gut microbiota composition and metabolism was observed at HADA_1 m, relative to both the LS and LA groups (Fig. 2a and b). This may be attributed to the adaptive homeostasis of the gut microbiota and fecal metabolism, as evidenced by the most pronounced alterations in the microbiota and metabolomic profiles during the initial month following descent from high to low altitudes.

We further delved into the intricate patterns and mechanisms underlying the hypoxia‒reoxygenation process in high-altitude populations through metabolic network analysis. Notably, L-glutamic acid (334 degrees), adenosine monophosphate (292 degrees), and oxoglutaric acid (268 degrees) presented the greatest number of associations (Fig. 3d). Mfuzz analysis stratified these DEMs into eight clusters based on their temporal trends, and KEGG pathway analysis of each cluster revealed diverse metabolic pathways impacted by hypoxia and reoxygenation (Fig. 3e). Notably, the 543 DEMs in Cluster C6 lacked significant pathway enrichment, suggesting that although metabolic changes in feces were pronounced within the first week after reoxygenation, they did not directly impact systemic metabolic functions. Conversely, DEMs in clusters C4 and C8, which were related primarily to energy metabolism, lipid metabolism, and oxidative stress (e.g., amino sugar and nucleotide sugar metabolism, linoleic acid metabolism, glycolysis/gluconeogenesis, necroptosis, prolactin signaling pathway, proximal tubule bicarbonate reclamation, biosynthesis of unsaturated fatty acids, folate biosynthesis, and bile secretion), were rapidly suppressed after reoxygenation. In contrast, DEMs in clusters C1, C5, and C7 involving vitamin B6 metabolism, purine metabolism, drug metabolism, that is, cytochrome P450, fatty acid biosynthesis, and steroid biosynthesis, were rapidly upregulated, highlighting their roles in neural regulation, energy metabolism, genetic information transfer, and drug metabolism. Finally, DEMs in cluster C3, which were associated primarily with energy metabolism (e.g., nicotinate and nicotinamide metabolism, citrate cycle [TCA cycle], glucagon signaling pathway, neomycin, kanamycin, and gentamicin biosynthesis), displayed a discernible trend only four months postreoxygenation. Metabolites in the cluster C2, which are enriched in the pathways of protein digestion and absorption, aminoacyl-tRNA biosynthesis, mineral absorption, D-amino acid metabolism, glycine, serine, threonine metabolism, and ABC transporters, were rapidly upregulated 1 month after reoxygenation and significantly downregulated after 4 months.

### Plasma metabolome characteristics during the hypoxia–reoxygenation period

**Fig 4:**
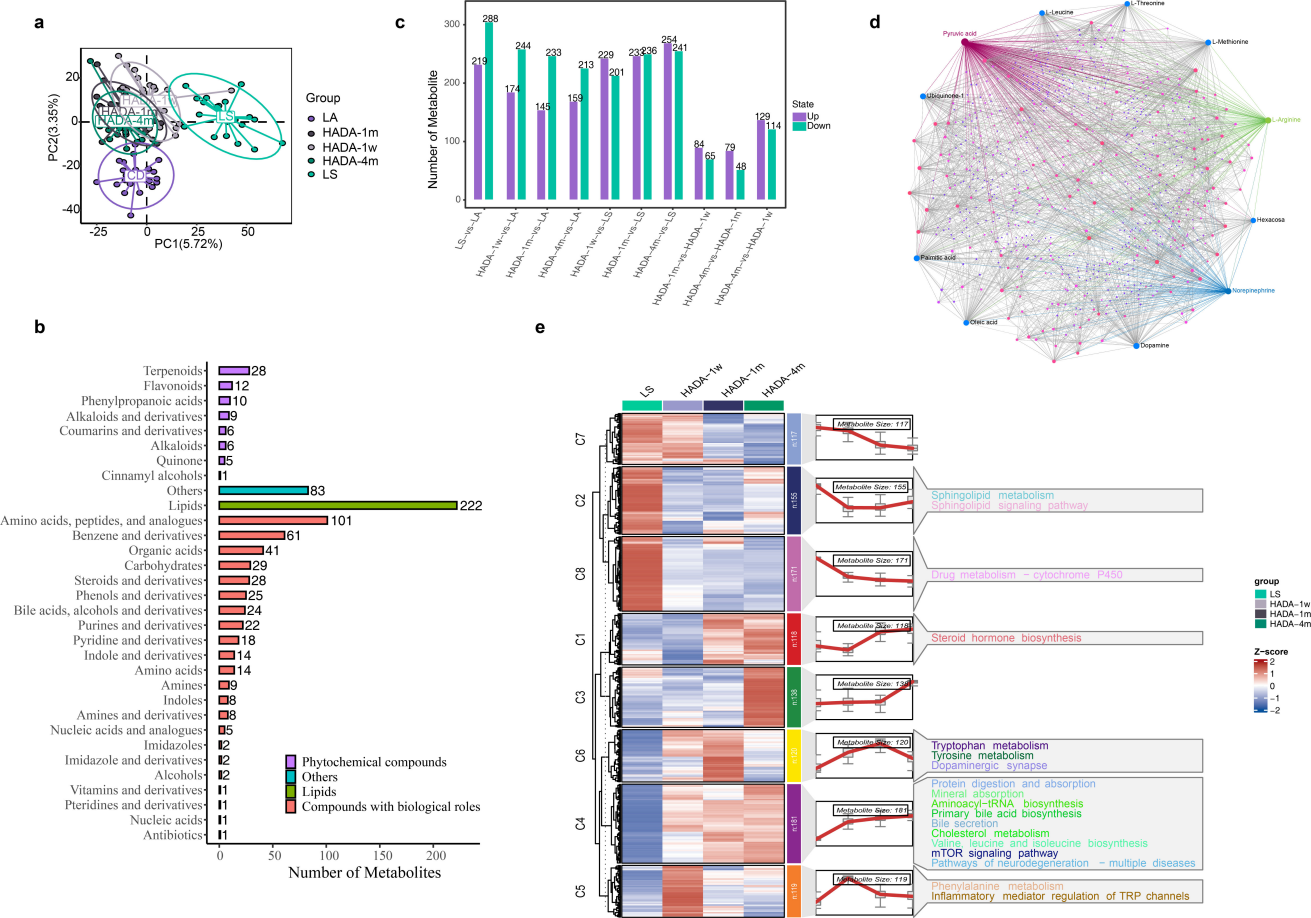
Overall characteristics of plasma metabolomics: (**a**) PLSDA analysis of plasma metabolomics in each group. (**b**) Classification of detected plasma metabolite types. Metabolites belonging to the lipid category are combined and displayed together. Metabolites not belonging to the categories of compounds with biological roles, phytochemical compounds, or lipids are represented as “others.” (**c**) Quantity of different metabolites in each comparison group. (**d**) Network analysis of total metabolites on the MetaboAnalyst website, where the size of the nodes represents the degree of correlation for each metabolite. The top 10 metabolites with the most relationship pairs are labeled with their names. Purple represents the network of pyruvic acid with other metabolites, blue lines represent the network of norepinephrine with other metabolites, and green lines represent the network of L-arginine with other metabolites. (**e**) Mfuzz analyzed the time trend of metabolites and divided them into clusters and enriched the metabolites contained in each cluster by KEGG pathway, only the significantly enriched pathways were shown in the figure (*P* < 0.05).

The application of PLSDA successfully distinguished the various groups, revealing significant differences in the plasma metabolome between the LS and LA groups (Fig. 4a). Notably, during reoxygenation, the metabolic profiles of the plateau population gradually converged toward those of the LA group, with HADA_4 m exhibiting the closest relationship to the LA group. This observation was further substantiated by the quantitative changes in differentially expressed metabolites, where the number of differentially expressed metabolites between high-altitude residents during reoxygenation and those residing at LA gradually decreased. Among the identified metabolites, lipids were the most abundant class (222 metabolites), followed by amino acids, peptides, and their analogs (101 metabolites) (Fig. 4b). Conversely, the number of differentially abundant metabolites between high-altitude residents during reoxygenation and those in the LS group gradually increased (Fig. 4c).

DEMs were identified on the basis of distinct groups, resulting in a total of 1119 DEMs present in at least one of the comparative groups. The metabolite‒metabolite network association analysis revealed that pyruvic acid (242 degrees), norepinephrine (112 degrees), and L-arginine (111 degrees) were the metabolites with the greatest numbers of associations (Fig. 4d).

Mfuzz analysis categorized the temporal trends of these metabolites into eight clusters, and KEGG pathway analysis was performed on the differentially abundant metabolites within each cluster, elucidating various metabolic pathways impacted by hypoxia and reoxygenation (Fig. 4e).

Notably, upon reoxygenation, the differentially abundant metabolites identified in C1, C2, and C8, which were related primarily to drug metabolism, the sphingolipid signaling pathway, sphingolipid metabolism, and drug metabolism, that is, cytochrome P450, and steroid hormone biosynthesis, were promptly suppressed. Conversely, the differentially abundant metabolites in C4 and C6, which encompassed various metabolic pathways and biological processes such as protein digestion and absorption, mineral absorption, aminoacyl−tRNA biosynthesis, primary bile acid biosynthesis, bile secretion, cholesterol metabolism, valine, leucine and isoleucine biosynthesis, mTOR signaling pathway, pathways of neurodegeneration—multiple diseases, tryptophan metabolism, tyrosine metabolism, and dopaminergic synapse, which are linked to nervous system function, amino acid metabolism, and cell growth and metabolism regulation, exhibited rapid upregulation after reoxygenation. Finally, the differentially abundant metabolites in C5 rapidly increased within the first week of reoxygenation, followed by a swift decline, impacting metabolic pathways related to inflammatory mediator regulation of TRP channels and phenylalanine metabolism.

### Changes in the intestinal flora and metabolism within 1 week after reoxygenation

**Fig 5:**
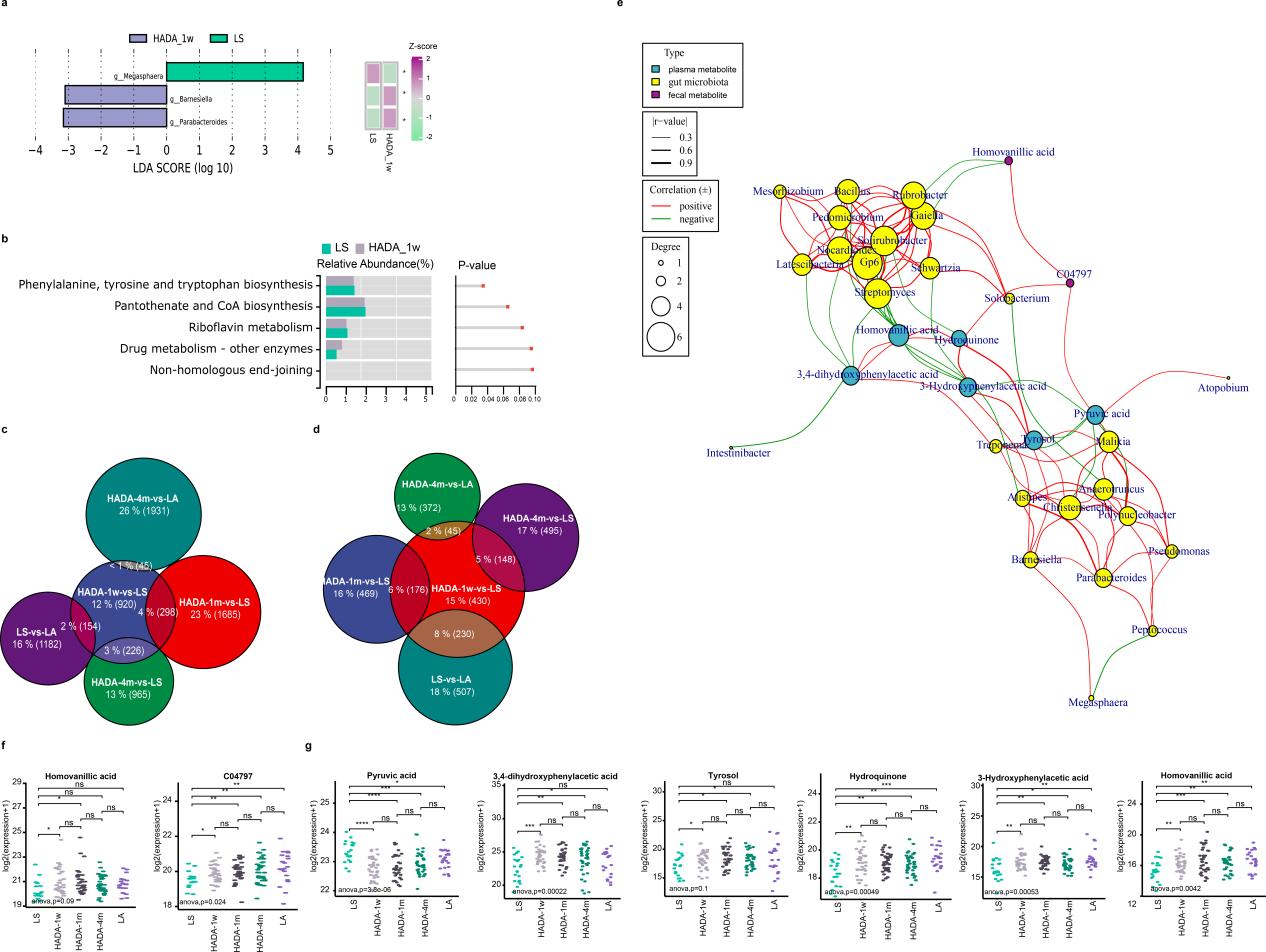
Analysis of the characteristics of intestinal flora, fecal metabolism, and plasma metabolism in the first week of reoxygenation in the plateau population. (**a**) LDA of LEfSe analysis (LDA > 3, *P* < 0.05). The heatmap shows the relative abundance of bacterial communities in the HADA_1 w and LS groups. The Kruskal-Wallis single-factor test was used to compare the relative abundance expression differences among multiple groups (*P < 0.05). (**b**) Based on the functional enrichment analysis of KEGG pathways predicted by PICRUSt2, the figure shows the top 5 KEGG pathways sorted by *P* value. (**c**) The number and proportion of differentially expressed metabolites in the HADA_1 w vs. LS fecal metabolome comparison also overlaps with differential expressions in other comparison groups. (**d**) The number and proportion of differentially expressed metabolites in the HADA_1 w vs. LS plasma metabolome comparison also overlaps with differential expressions in other comparison groups. (**e**) Spearman correlation analysis of differentially expressed fecal and plasma metabolites involved in tyrosine metabolism (HADA_1 w vs. LS) with bacterial genera showing significant differential abundance (*r* ≧ 0.3, *P* < 0.05). (**f**) Boxplot illustrates the differential expression of metabolites involved in tyrosine metabolism between the HADA-1w and LS groups in the fecal metabolome (ns = no significance, *P < 0.05, **P < 0.01, ***P < 0.001). (**g**) The boxplot illustrates the differential expression of metabolites involved in tyrosine metabolism between the HADA_1 w and LS groups in the plasma metabolome (ns = no significance, *P < 0.05, **P < 0.01, ***P < 0.001).

To elucidate the gut microbiome and metabolic alterations associated with reoxygenation, we compared the gut microbiota and metabolites between the HADA_1 w and LS groups. Our findings revealed significant increases in the abundances of *Barnesiella* and *Parabacteroides* in the HADA_1 w group, whereas the abundance of *Megasphaera* notably decreased (Fig. 5a). These changes may play pivotal roles in adapting to reoxygenation. Furthermore, the random forest analysis underscored the discriminatory power of the *Barnesiella*, *Parabacteroides*, and *Megasphaera* genera (Fig. S4a).

A comprehensive analysis of differential fecal metabolites revealed a total of 920 unique entities in the HADA_1 w versus LS comparison, with significant overlaps observed: 154 overlapping DEMs in the LS and LA comparison, 298 overlapping DEMs in the HADA_1 m and LS comparison, 226 overlapping DEMs in the HADA_4 m and LS comparison, and 130 overlapping DEMs in the HADA_4 m and LA comparison (Fig. 5c). Similarly, in the plasma metabolome, 430 differentially expressed metabolites were identified in the HADA_1 w versus LS comparison, exhibiting overlaps of 230 DEMs in the LS and LA comparison, 176 DEMs in the HADA_1 m and LS comparison, 148 DEMs in the HADA_4 m and LS comparison, and 45 DEMs in the HADA_4 m and LA comparison (Fig. 5d).

The observed overlap and distinct differences in DEMs between the initial week of reoxygenation and subsequent time points indicated that although substantial initial metabolic adjustments occurred, these initial changes in both the fecal and plasma metabolomes gradually stabilized over time. The persistence of subsets of DEMs suggests that certain metabolic pathways in highlanders may remain altered even after prolonged adaptation to low altitudes, reflecting either ongoing adaptation or lingering effects of prior high-altitude exposure. Furthermore, the persistence of distinct metabolic signatures between highlanders and lowlanders even after 4 months of acclimatization underscores the necessity for further investigation into the environmental factors contributing to these differences.

To deepen our understanding of metabolic shifts in highlanders during hypoxic-reoxygenation, we employed Mfuzz analysis, which provided valuable insights into the temporal dynamics of metabolic adaptation. Compared with those of the LS group, the fecal metabolome of HADA-1w exhibited rapid increases in metabolites associated with clusters 1, 5, and 7, whereas the plasma metabolome displayed similar surges in clusters 4, 5, and 6 (Fig. 3e and 4e). Clusters C6 in Fig. 4e converged on tyrosine metabolism in KEGG pathway analyses. In the comparison group of HADA_1 w vs LS, the DA score in the KEGG analysis shows that tyrosine metabolism is upregulated in both plasma (DA score = 0.667) and fecal (DA score = 1) metabolisms, indicating a potential metabolic link or functional similarity.

The correlation network analysis between the gut microbiota and plasma and fecal tyrosine metabolisms revealed significant associations between the abundances of 23 bacterial genera and the expression levels of two fecal metabolites (homovanillic acid and C04797) and six plasma metabolites (pyruvic acid, 3,4-dihydroxyphenylacetic acid, tyrosol, hydroquinone, 3-hydroxyphenylacetic acid, and homovanillic acid) within 1 week of exposure to the plains in the high-altitude populations (Fig. 5e). These bacterial genera may contribute to the host’s adaptation to hypoxic environments at high altitudes by producing or consuming specific metabolites, including intermediates involved in tyrosine metabolism. Notably, except for pyruvic acid, which significantly decreased at HADA_1 w in plasma, all the other mentioned metabolites displayed notable increases (Fig. 5f and g). Notably, 3-hydroxyphenylacetic acid in plasma emerged as a potential biomarker via random forest analysis (Fig. S4c), exhibiting a positive correlation with *Barnesiella* abundance (*r* = 0.36, *P* = 0.012) and an inverse correlation with Gp6 (*r* = −0.38, *P* = 0.009), Schwartzia (*r* = −0.31, *P* = 0.033), and *Streptomyces* (*r* = −0.35, *P* = 0.016) (Fig. 5e).

The functional prediction of the gut microbiota revealed significant differences in phenylalanine, tyrosine, and tryptophan biosynthesis between high-altitude residents during reoxygenation and LS baseline individuals (*P* < 0.05) (Fig. 5b). Despite this, the FDR value of 1 implies that these findings may lack statistical robustness. However, tyrosine metabolism seems to be regulated by the gut microbiota, their metabolites, and plasma metabolism. As illustrated in Fig. 5e, there is a significant association between bacterial genera that change notably during the first week of reoxygenation and differentially abundant metabolites involved in tyrosine metabolism, implying its activation and concurrent modulation may be the gut microbiota and blood components.

To explore the relationships among the gut microbiota, fecal and plasma metabolomes, and HADAS scores during the first week of reoxygenation in high-altitude residents in greater detail, we conducted a correlation analysis between the multiomics data and HADAS scores. Our findings revealed significant associations between the expression levels of six plasma metabolites and one bacterial genus (*Rothia*) with HADAS scores (│*r*│ > 0.5, *P* < 0.05) (Fig. S5a). Notably, these biomarkers also exhibited distinct differences when participants were stratified on the basis of the presence or absence of HADAS symptoms, with a HADAS score threshold of 5 (Fig. S5b). Specifically, hypoxanthine, which is involved in purine metabolism, and pyridoxine, which is involved in vitamin B6 metabolism, suggested pivotal roles for these metabolic pathways in the body’s adaptation to reoxygenation at lower altitudes. Consequently, these biomarkers may represent potential therapeutic targets for preventing HADAS symptoms.

### Characteristics of oxidative stress in the gut microbiota and metabolism in response to reoxygenation

**Fig 6:**
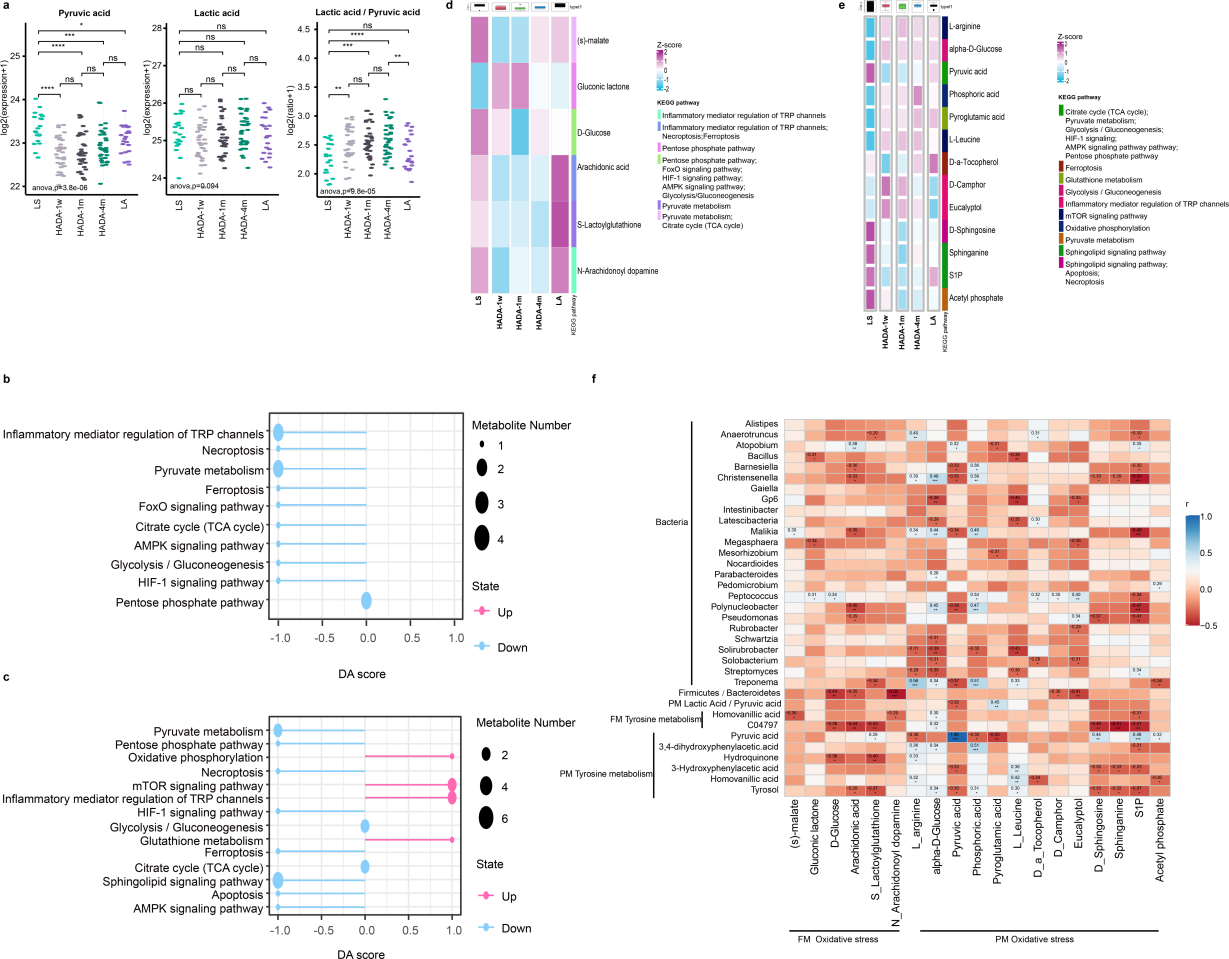
Changes in oxidative stress levels after 1 week of reoxygenation among high-altitude residents. (**a**) Boxplot displaying differential expression of plasma metabolites lactic acid, pyruvic acid, and the lactic acid/pyruvic acid ratio (ns  =  no significance, *P < 0.05, **P < 0.01, ***P < 0.001). (**b**) Differential abundance (DA) scores of oxidative stress-related pathways from functional enrichment analysis of differentially expressed fecal metabolites in the HADA_1 w vs. LS comparison group. (**c**) DA scores of oxidative stress-related pathways from functional enrichment analysis of differentially expressed plasma metabolites in the HADA_1 w vs. LS comparison group. A DA score of 1 indicates an upregulation trend for all identified metabolites in the pathway, whereas −1 indicates a downregulation trend. The length of the line segment represents the absolute value of the DA score, and the size of the dot at the end of the line segment represents the number of metabolites in the pathway. (**d**) Heatmap of differentially expressed fecal metabolites involved in oxidative stress-related pathways from Fig. 6b. (**e**) Heatmap of differentially expressed plasma metabolites involved in oxidative stress-related pathways from Fig. 6c. (**f**) Spearman correlation analysis between oxidative stress-related metabolites and differentially expressed bacterial genera (*P* < 0.05), Firmicutes/Bacteroidetes ratio, lactic acid/pyruvic acid ratio, and Tyrosine metabolism metabolites in the HADA_1 w vs. LS comparison group (*P < 0.05, **P < 0.01, ***P < 0.001). PM: plasma metabolome. FM: fecal metabolome.

Pyruvic acid, a marker of hypoxia, was the key node in our network analysis (Fig. 4d and 5g). Its decrease after reoxygenation indicated its crucial role in oxidative stress. Hypoxia shifts cells to anaerobic metabolism, increasing pyruvic acid production, whereas oxidative stress further affects pyruvic acid metabolism. The lactate/pyruvate ratio reflects the cytosolic NAD+/NADH balance and is associated with oxidative stress (15). In our study, both lactate and pyruvic acids were detected in plasma, with pyruvic acid being most abundant in the LS group and least abundant in the HADA_1 w group (Fig. 6a). Lactate abundance was greater in plateau dwellers but was not significantly different after reoxygenation. Compared with normoxic conditions, the lactate/pyruvate ratio is lower in chronically hypoxic hearts (16). The lactate/pyruvate ratio was lowest in the LS group (16) and increased after reoxygenation (Fig. 6a), reflecting the sharp change of oxidative stress.

To further evaluate the oxidative stress characteristics during reoxygenation, we conducted DA score analysis on oxidative stress-related metabolic pathways enriched with differentially abundant metabolites in the HADA_1 w vs. LS comparison group. In the fecal metabolome, oxidative stress-related pathways were consistently downregulated (Fig. 6b). Conversely, in the plasma metabolome, pathways such as oxidative phosphorylation, glutathione metabolism, the mTOR signaling pathway, and inflammatory mediator regulation of TRP channels were upregulated, whereas oxidative stress-related pathways were downregulated (Fig. 6c). These findings suggest a dynamic adaptation in oxidative stress levels during the first week of reoxygenation among high-altitude residents. Notably, the opposing trend observed in inflammatory mediator regulation of TRP channels between the plasma and fecal metabolomes indicated distinct metabolic environments, inflammatory mediator mechanisms, or TRP channel functional states in response to reoxygenation.

To gain insight into the relationships among oxidative stress, metabolism, and the gut microbiota, we visualized, with heatmaps, the expression levels of these oxidative stress-related fecal metabolites (Fig. 6d) and plasma metabolites (Fig. 6e) across reoxygenation periods. Substantial differences were observed between high- and low-altitude individuals, with significant changes occurring during the first week of reoxygenation, followed by a relatively stable state. However, the differences persisted compared with those of the low-altitude individuals. Notably, plasma L-arginine, D-erythrosphingosine 1-phosphate (S1P), and alpha-D-glucose were identified as potential biomarkers in random forest analysis, indicating their roles in cell signaling, proliferation, migration, apoptosis, and energy metabolism. These findings suggest an active response during the first week of reoxygenation to mitigate oxidative damage.

Spearman’s correlation analysis revealed a positive correlation between the oxidative stress marker lactic acid/pyruvic acid ratio and the plasma metabolite pyroglutamic acid (Fig. 6f). Notably, greater numbers of significantly altered bacterial genera were significantly correlated with oxidative stress-related plasma metabolites. Additionally, the F/B ratio, which is a gut homeostasis marker, was negatively correlated with fecal D-glucose, arachidonic acid, N-arachidonoyl dopamine, plasma D-camphor, and eucalyptol, indicating that both fecal and plasma metabolism during the first week of reoxygenation may influence gut microbiota homeostasis by modulating the oxidative stress status. Furthermore, metabolites associated with the activated tyrosine metabolism pathway (in both plasma and feces) during the first week of reoxygenation were significantly negatively correlated with oxidative stress-related metabolites, suggesting a potential role of tyrosine metabolism in oxidative stress resistance during the reoxygenation phase among high-altitude residents.

### Metabolic changes in high-altitude residents after prolonged reoxygenation

**Fig 7:**
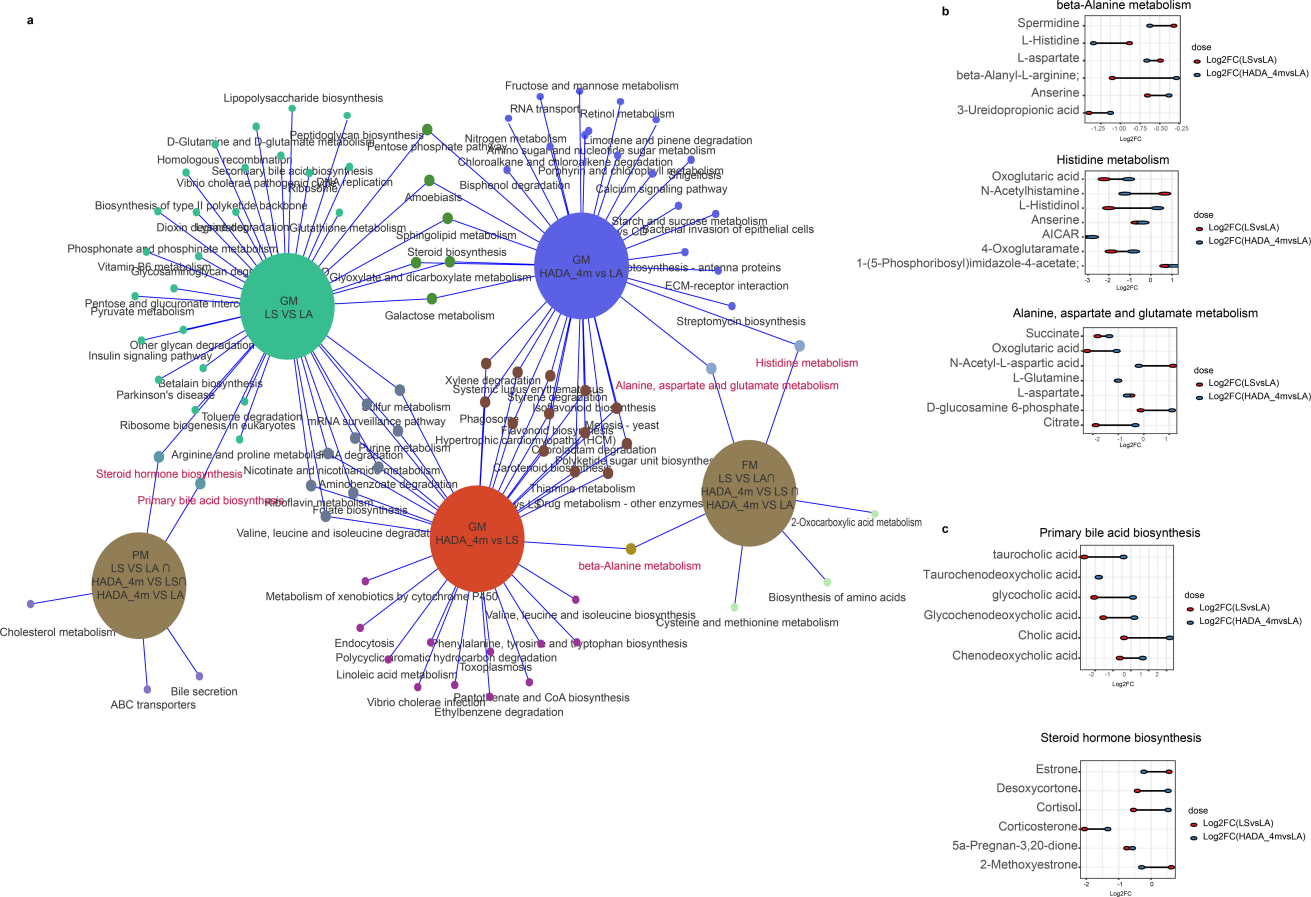
Multi-omics characterization of metabolic differences in high-altitude residents after prolonged reoxygenation. (**a**) Functional enrichment analysis based on KEGG predicted by PICRUSt2 from gut microbiota in high-altitude residents during prolonged reoxygenation (HADA_4 m) compared with their baseline at high altitude and to low altitude residents, as well as enrichment in fecal and plasma metabolomes. Green circles represent the LS vs. LA comparison group. Orange circles represent the HADA_4 m vs. LS comparison group. Purple circles represent the HADA_4 m vs. LA comparison group. Brown circles represent the combined overlapping pathways from the various comparison groups in both plasma and fecal metabolomes. (**b**) Dumbbell plot showing the log2FC values of fecal metabolites involved in alanine, aspartate, and glutamate metabolism, Histidine metabolism, and beta-alanine metabolism. Red dots represent log2FC(LS vs. LA), and blue dots represent log2FC(HADA_4 m vs. LA). (**c**) Dumbbell plot showing the log2FC values of plasma metabolites involved in Steroid hormone biosynthesis and Primary bile acid biosynthesis. Red dots represent log2FC(LS vs. LA), and blue dots represent log2FC(HADA_4 m vs. LA).

To elucidate the molecular mechanisms underlying acclimatization symptoms following prolonged reoxygenation, differential enrichment analyses were conducted among the LS, HADA_4 m, and LA groups. In the fecal metabolome, six shared differential metabolic pathways were identified across the three comparisons: biosynthesis of amino acids; 2-oxocarboxylic acid metabolism; alanine, aspartate and glutamate metabolism; histidine metabolism; cysteine and methionine metabolism; and beta-alanine metabolism. These findings suggest significant adjustments in amino acid demand and utilization during the transition from high altitude to plain (Fig. S6a). Notably, alterations in alanine, aspartate and glutamate metabolism, histidine metabolism, and beta-alanine metabolism were also predicted in the gut microbiota comparisons (Fig. 7a). Following long-term reoxygenation, the majority of metabolites involved in thesethree pathways in high-altitude residents were downregulated, with the exceptions of L-histidinol, 1-(5-phosphoribosyl)imidazole-4-acetate, and D-glucosamine 6-phosphate, which were upregulated compared with those in low-altitude residents (Fig. 7b).These alterations may reflect the long-term influence of high-altitude exposure on amino acid synthesis and metabolism, as well as the metabolic adjustments related to protein synthesis, energy metabolism, and neurotransmitter synthesis after reoxygenation. Previous studies have reported that under hypoxic conditions, the level of oxidative stress within the body increases and that amino acid metabolism may contribute to cellular resilience against damage by enhancing antioxidant capabilities (17， 18,^18^). Furthermore, research has indicated that bile acid synthesis and excretion may be regulated under hypoxia, thereby influencing lipid metabolism and inflammatory responses (19). Hypoxia stimulates the adrenal glands to secrete steroid hormones, increasing the levels of anti­inflammatory hormones, which can mitigate tissue damage and inflammation induced by hypoxia to a certain extent (20). In the present study, the observed changes in amino acid metabolism may impact antioxidant synthesis (21), whereas alterations in steroid hormone (22) and bile acid metabolism (23) may be intricately linked to the modulation of inflammatory responses.

In the plasma metabolome, five common differential metabolic pathways were identified across the three comparison groups: cholesterol metabolism, bile secretion, steroid hormone biosynthesis, primary bile acid biosynthesis, and ABC transporters (Fig. S6b). Notably, alterations in steroid hormone biosynthesis and primary bile acid biosynthesis were also predicted in the gut microbiota comparison between the LS (high-altitude residents in Lhasa) and LA (low-altitude residents) groups. Additionally, alterations in these two pathways were further predicted in the gut microbiota comparison between the HADA_4 m (high-altitude residents after 4 months at low altitude) and LA groups (Fig. 7a). Most plasma metabolites associated with these pathways presented increased expression following reoxygenation (Fig. 7b). The significant trends observed in the plasma metabolome suggest that the reoxygenation process may activate the endocrine and digestive system functions of the body. Collectively, these pathways regulate the body’s adaptation to new environments by influencing energy metabolism, protein synthesis, neurotransmitter transmission, endocrine regulation, and immune responses.

KEGG functional prediction of the gut microbiota revealed that energy metabolism, the antioxidant stress response, and immune regulation may be associated with the specific physiological demands of high-altitude residents (Fig. 7a). Under reoxygenated conditions, the gut microbiota of high-altitude residents underwent adaptive changes, resulting in significant differences in key life processes, such as energy supply, antioxidant defense, and DNA repair, compared with their state at high altitude.

## DISCUSSION

Four months of reoxygenation is insufficient to reverse the physiological alterations induced by hypoxic exposure, as the adaptation process to the high-altitude environment has already instigated profound physiological changes within the body, making it difficult for individuals to return to a state comparable with those residing at low altitude (24, 25). At 1 week, 1 month, and 4 months after reoxygenation among individuals residing at high altitudes, blood parameter analysis revealed significant alterations in erythrocyte-related indices. Notably, HGB, a diagnostic marker for high-altitude polycythemia, consistently decreased during the reoxygenation phase, with no significant difference from that in low-altitude populations at 4 months. Microbiome, fecal metabolome, and plasma metabolome analyses revealed substantial disparities between high-altitude and low-altitude populations in terms of amino acid metabolism, lipid metabolism, energy metabolism, immunity, cofactors, and vitamin metabolism. Furthermore, the functional profiles of high-altitude residents differed significantly from their own profiles during high-altitude residence after reoxygenation. Although metabolic changes at 1 week after reoxygenation largely recovered to baseline levels by 4 months (compared with those in the low-altitude control group), numerous bacterial genera and metabolites remained at distinct levels of abundance compared with those in low-altitude populations.

### Dynamic effects of plateau hypoxia and reoxygenation on intestinal microbiota diversity

Changes in individual altitude exposure elicit alterations in the diversity, abundance, and composition of the gut microbiota (26). Among high-altitude residents, the highest alpha diversity of the gut microbiome was observed 1 month after reoxygenation, reflecting dynamic changes in the abundance and diversity of the gut microbiota during hypoxia–reoxygenation (27). Notably, significant shifts in gut microbiome beta-diversity emerged only after 4 months of reoxygenation, suggesting a relatively stable intestinal microecosystem among high-altitude residents that enables better adaptation to external environmental changes and maintains normal intestinal function. The absence of marked alterations in the F/B ratio further underscores the resilience of the gut microbiota to altitude variations. This stability may be associated with the increased oxygen-carrying capacity of erythrocytes in high-altitude populations (28), potentially mitigating the adverse effects of hypoxia–reoxygenation on the gut microbiota. Nonetheless, at 1 week after reoxygenation, the gut microbiota, fecal metabolome, and plasma metabolome displayed sensitivity to reoxygenation, as evidenced by alterations in microbiota abundance and metabolite expression levels compared with those in the LS group.

In the context of gut microbiota data, prolonged hypoxic exposure was found to alter intestinal barrier permeability and the composition of the gut microbiota. Specifically, the abundance of the genus *Alloprevotella* increased in the LS group, and *Alloprevotella* metabolites, especially short-chain fatty acids (SCFAs), display antioxidant activities, assisting the host against oxidative stress by modulating immunity and reducing inflammation (29). Within the first week of reoxygenation, the *Barnesiella* and *Parabacteroides* abundances increased significantly, whereas the *Megasphaera* abundance decreased. *Parabacteroides* may aid in the defense against gut infection, and *Barnesiella* may lower inflammation and enhance metabolic health (30). *Megasphaera*, through fermentation of lactate to SCFAs, maintains the gut balance and may impact oxidative stress (31).

These results indicate that the gut microbiota of individuals residing at high altitudes undergoes rapid alterations in intestinal barrier function, immune response, inflammation, and metabolism in response to changes in altitude and diet during the reoxygenation phase. Furthermore, the gut microbiota not only impacts host health through direct metabolic activities but also potentially contributes to maintaining physiological homeostasis by modulating inflammatory and oxidative states.

### Restructuring of oxidative stress and antioxidant mechanisms during the initial phase of reoxygenation

Hypoxia-induced oxidative stress at high altitudes constitutes a pivotal pathogenic mechanism underlying the development of high-altitude diseases (32, 33). Oxidative stress plays a significant role in the response to hypoxia and reoxygenation (33). During the first week of reoxygenation, a marked decrease in pyruvate levels was observed, with lactate and pyruvate, which are mediated by hormonal ROS signaling pathways, contributing significantly to oxidative stress resistance (34). Plasma L-arginine, S1P, and alpha-D-glucose were implicated in oxidative stress-related pathways during the first week of reoxygenation and identified as biomarkers of the reoxygenation process. L-arginine has favorable effects on hypoxic injuries in clinical settings (35), and its metabolic products serve as pivotal components in various biological processes in mammals and microorganisms, playing crucial roles in host-microbe interactions (36). Prolonged residence at high altitudes leads to reduced plasma glucose levels (37). S1P, a component of the sphingolipid signaling pathway, has been identified as an intracellular hypoxia-responsive biological lipid that facilitates erythrocyte glycolysis and O2 transport (38). Its expression during reoxygenation is postulated to be directly influenced by erythrocyte-related indices in blood, such as RBC and HGB (39). The decreased plasma abundance of S1P may indicate a reduction in erythrocyte turnover or a shift in erythrocyte metabolism as part of the adaptive response to reoxygenation.

At high altitudes, the hypoxic environment leads to an increase in ROS within mitochondria and other cellular organelles (32). Reoxygenation, the subsequent restoration of oxygen levels in the blood, promptly enhances cellular metabolic activity, accelerating the elimination of ROS and resulting in a notable reduction in oxidative stress levels (40). The present study revealed a notable increase in the levels of lactic acid/pyruvic acid during the reoxygenation phase, which, coupled with the downregulation of oxidative stress-related metabolic pathways and upregulation of antioxidant pathways after 1 week of reoxygenation, suggests a significant alteration in oxidative stress levels and an increase in antioxidant levels within the initial week among high-altitude populations. Nevertheless, further investigation is imperative to elucidate the precise underlying mechanisms and implications of oxidative stress alterations in these populations, along with the identification of safe strategies to modulate their levels. *Lactobacillus johnsonii* YH1136 alleviates intestinal barrier impairment and inflammation in hypoxic mice at high altitudes, preserving intestinal integrity and mitigating systemic oxidative stress (41). An oligofructose prebiotic has been shown to safeguard human umbilical vein endothelial cells from hypoxia/reoxygenation damage, likely because of its antioxidant and immunomodulatory properties (42). Furthermore, fecal microbiota transplantation (FMT) has been demonstrated to significantly improve the gut microbiota composition in animal models, thereby enhancing antioxidant defenses and exerting protective effects against oxidative damage induced by hypoxia‒reoxygenation, ultimately reducing oxidative stress levels (43, 44). By modulating the gut microbiota, decreasing oxidative stress, and augmenting antioxidant capacity, the trifecta of probiotics, prebiotics, and FMT represents a promising therapeutic strategy for managing hypoxia‒reoxygenation injuries at high altitudes.

### Activation of tyrosine metabolism and antioxidant stress potential during the initial phase of reoxygenation

Tyrosine metabolism has been implicated in myocardial ischemia‒reperfusion (45) and pulmonary hypertension (46). During acute hypoxemia induced by high-altitude exposure, significant alterations in the human gut microbiota significantly modulate tyrosine metabolism, thereby influencing the body’s adaptability to high-altitude stress (47). Our study’s correlation network analysis of gut microbiota with plasma and fecal tyrosine metabolism, alongside functional prediction analyses of the gut microbiota and KEGG pathway enrichment of DEMs, revealed that during the first week of reoxygenation in high-altitude populations, tyrosine metabolism is associated with gut microbiota, fecal, and plasma metabolism, exhibiting significant metabolite differences compared with their high-altitude levels throughout the reoxygenation phase. Furthermore, certain gut microbes can ferment tyrosine into potentially toxic compounds such as ammonia and amines, impacting host health (48). Furthermore, a previous study by Pi et al. (49) demonstrated that treatment with a cocktail of antibiotics (AGM) significantly altered specific gut microbiota and markedly increased serum tyrosine concentration in growing pigs, although it reduced the efficiency of tyrosine utilization. Based on the findings from Murray et al. (48) and Pi et al. (49), we hypothesize that tyrosine metabolism plays a pivotal role during reoxygenation, particularly within the first week. Plasma and fecal tyrosine metabolism were activated during this initial week, whereas gut microbiota-mediated phenylalanine, tyrosine, and tryptophan biosynthesis were suppressed. Notably, the relevant metabolites were negatively correlated with oxidative stress metabolites, suggesting that tyrosine metabolism is involved in antioxidant defense. These findings have important implications for understanding the complex interactions between gut microbiota and tyrosine metabolism during reoxygenation in high-altitude populations. However, further studies are needed to confirm these observations and explore the underlying mechanisms in more detail.c

### Far-reaching impacts of long-term high-altitude exposure and reoxygenation on lipid metabolism, the endocrine system, and amino acid metabolism

The metabolic consequences of prolonged high-altitude exposure persist even after 4 months of reoxygenation, failing to align with those of individuals residing at low altitude. Significant disparities were observed in plasma metabolic pathways related to cholesterol metabolism, bile secretion, steroid hormone biosynthesis, and primary bile acid biosynthesis between high-altitude dwellers and low-altitude individuals, both during high-altitude exposure and after prolonged reoxygenation at low altitude. These disparities, compared with the metabolic profiles at high altitudes, further underscore the profound and long-lasting effects of chronic hypoxia and altitude changes on lipid metabolism (50), immunological homeostasis (51, 52), and the endocrine system. These alterations may facilitate adaptation to new environmental conditions by modulating inflammatory responses, energy metabolism, and substrate utilization. Additionally, persistent differences between high-altitude and low-altitude populations in fecal metabolic pathways involving amino acid biosynthesis, 2-oxocarboxylic acid metabolism, alanine, aspartate, and glutamate metabolism, histidine metabolism, cysteine and methionine metabolism, and beta-alanine metabolism suggest a close link between gut microbiota modulation and disparities in amino acid metabolism (53). The intricate adjustments in amino acid metabolism during reoxygenation among high-altitude residents may be crucial for restoring protein synthesis, energy supply, and neurotransmission functions in a low-altitude environment (54, 55).

Phenylalanine metabolism, the pentose phosphate pathway (12), sphingolipid metabolism (56), the tricarboxylic acid (TCA) cycle (57), glycolysis, purine degradation (58), alanine, aspartate, and glutamate metabolism, purine metabolism, and pyruvate metabolism (59) have been reported to undergo significant alterations in response to hypoxia, normoxia, and hypoxia/reoxygenation. Consistent with these findings, our study highlights the pivotal roles of these pathways during hypoxia/reoxygenation and prolonged reoxygenation in high-altitude populations.

### The synergistic effect of plasma metabolites and the intestinal flora on alleviating deacclimatization symptoms

The initial week of deacclimatization may be characterized by a concerted regulation of plasma metabolism and the gut microbiota. Notably, the plasma levels of pyridoxine, hypoxanthine, and *Rothia* bacteria were significantly correlated with HADAS scores during the first week of reoxygenation. Moreover, these biomarkers displayed marked differences between symptomatic and asymptomatic individuals undergoing deacclimatization during this period. Previous studies have reported reduced pyridoxine content in HK-2 cells under hypoxic conditions (60), whereas hypoxanthine accumulates during hypoxia in crucian carp and decreases upon reoxygenation (58). However, *Rothia* bacteria are known to be more abundant in mild cases of acute exacerbations of chronic obstructive pulmonary disease (61) but significantly less abundant in patients with osteosarcoma (62). The alterations observed in these metabolites and bacterial genera after reoxygenation could be attributed to changes in altitude, with individual variations in metabolite levels and bacterial abundances contributing to the diversity of deacclimatization symptoms. Further investigation into the underlying mechanisms is warranted.

Residents who have resided at high altitudes for extended periods may have developed adaptive changes in their metabolism and gut microbiota to cope with hypoxic conditions. However, upon exposure to normoxic environments, these adaptive responses may not fully revert within 4 months of reoxygenation. This could explain why some individuals who have resided at high altitudes continue to face health risks after relocating to lower altitudes. The present study aims to gain a deeper understanding of the adaptive processes involved in hypoxia and reoxygenation at high altitudes by examining metabolic and physiological mechanisms. This investigation not only enhances our understanding of these processes but also lays a theoretical foundation for the development of novel therapeutic strategies to prevent and treat oxidative damage. Nevertheless, further medical research is warranted at both the basic and clinical levels to elucidate the mechanisms underlying metabolite changes and their implications for human health.

### Conclusion

In conclusion, this study provides profound insights into the physiological adaptations of high-altitude residents to hypoxic and reoxygenated environments. During the first week of reoxygenation, significant alterations in the gut microbiota composition, fecal metabolism, and plasma metabolism were observed among the high-altitude inhabitants. As the duration of exposure to the plains increased, these disparities gradually diminished compared with those residing at low altitudes; however, distinct differences persisted even after 4 months of reoxygenation. Furthermore, the gut microbiota and fecal metabolites displayed temporal adaptability to the reoxygenation environment, with the first month of reoxygenation representing a crucial juncture for the gut microbiota and its metabolic adaptation in high-altitude residents. The bacterial genera *Parabacteroides, Barnesiella,* and *Megasphaera* play fundamental roles in the adaptation of high-altitude residents to hypoxic and reoxygenation environments. Additionally, they regulate physiological functions during reoxygenation by engaging in metabolic interactions within the host. The plasma metabolites L-arginine, S1P, and alpha-D-glucose played pivotal roles in mitigating oxidative stress during reoxygenation. Additionally, the activation of tyrosine metabolism in both feces and plasma during the reoxygenation phase suggested its potential involvement in antioxidant responses among high-altitude residents. Overall, this study highlights the pivotal roles of gut microbiota, fecal metabolome, and plasma metabolome in high-altitude adaptation to low-altitude environments, emphasizing their interconnectedness in maintaining physiological homeostasis.

## Data Availability

The data that support the findings of this study have been deposited into OMIX and GSA-human of China National Center for Bioinformation (CNCB) with accession number PRJCA024705: OMIX006101, OMIX006103, and HRA007065. Code and detailed information are available on github (https://github.com/langlibaitiaoshuafeidao/Exposure-to-high-altitude-leads-to-disturbance-of-host-metabolic-homeostasis/tree/main/R_CODE).

## SUPPLEMENTAL MATERIAL

Supplemental Figures msystems.01347-24-s0001.docx Figures S1 to S6.

Supplemental Tables msystems.01347-24-s0002.xlsx Tables S1 to S4.

ASM does not own the copyrights to Supplemental Material that may be linked to, or accessed through, an article. The authors have granted ASM a non-exclusive, world-wide license to publish the Supplemental Material files. Please contact the corresponding author directly for reuse.
