# Supplementary material for: Exposure to high altitude leads to disturbances in host metabolic homeostasis: study of the effects of hypoxia-reoxygenation and the associations between the microbiome and metabolome
Source: mSystems. 2025 Apr 16;10(5):e01347-24. doi: 10.1128/msystems.01347-24 (PMC12090774; doi:10.1128/msystems.01347-24)
Supplement: Supplemental Figures — Figures S1 to S6. [file msystems.01347-24-s0001.docx]

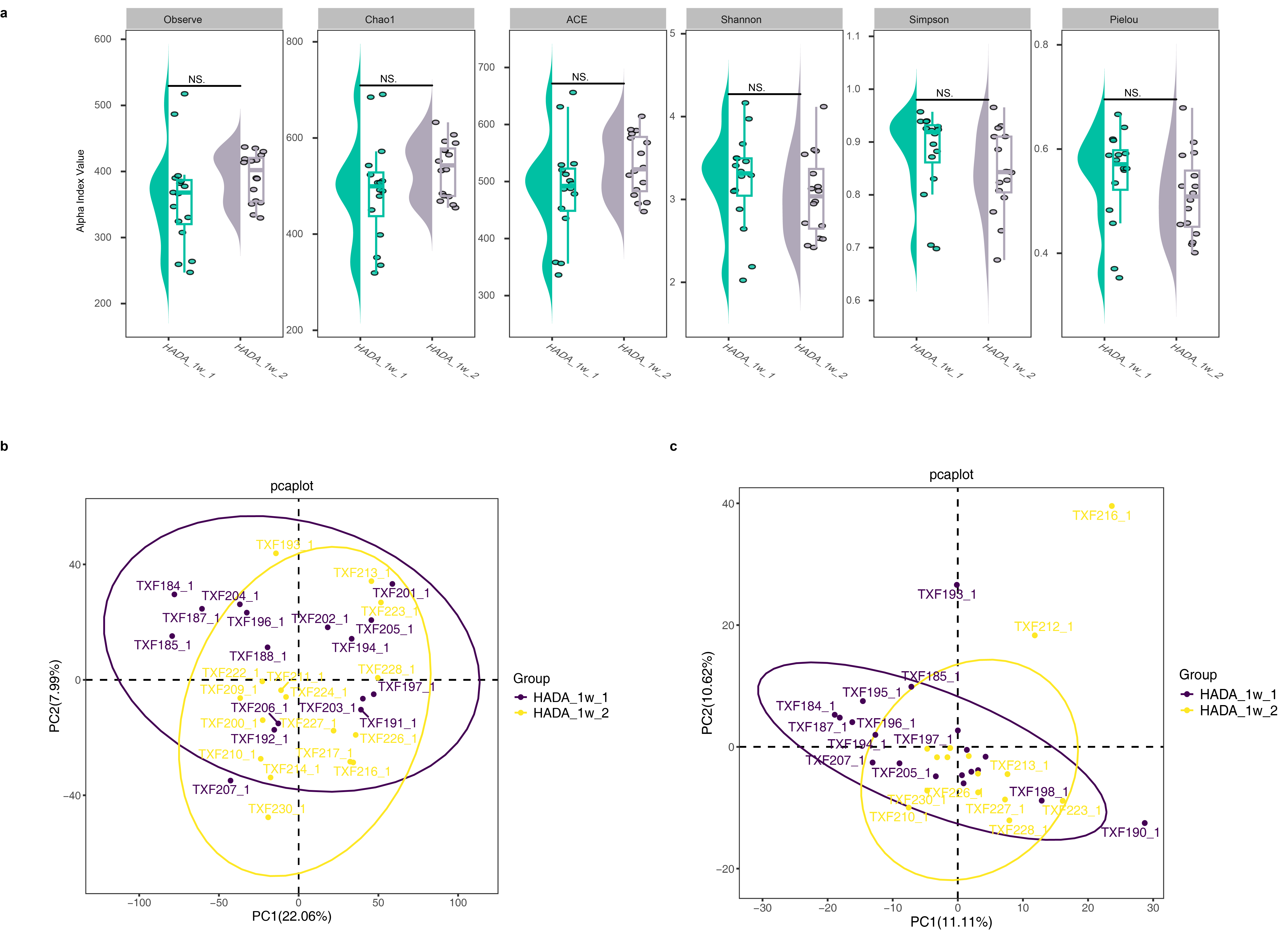


Figure S1. Analysis of differences in omics data between high-altitude populations recruited at high altitude (HADA_1w_1) and those recruited at plain and then ascended to high altitude (HADA_1w_2). a. Comparison of gut microbiota alpha diversity between HADA_1w_1 and HADA_1w_2. b. PCA analysis of fecal metabolome for HADA_1w_1 and HADA_1w_2 groups. c. PCA analysis of plasma metabolome for HADA_1w_1 and HADA_1w_2 groups.



Figure S2. Assessment of blood routine data and redundancy analysis based on Bray-Curtis distance (dbRDA). (a) gut microbiota, (b) fecal metabolome, and (c) plasma metabolome with blood routine indices. The percentages in parentheses indicate the variance explained by each axis. (d) Individual effects of blood routine indices on different omics datasets. The individual effect of each blood routine index on metabolites or intestinal microbiota was calculated using the 'rdaca.hp' package.


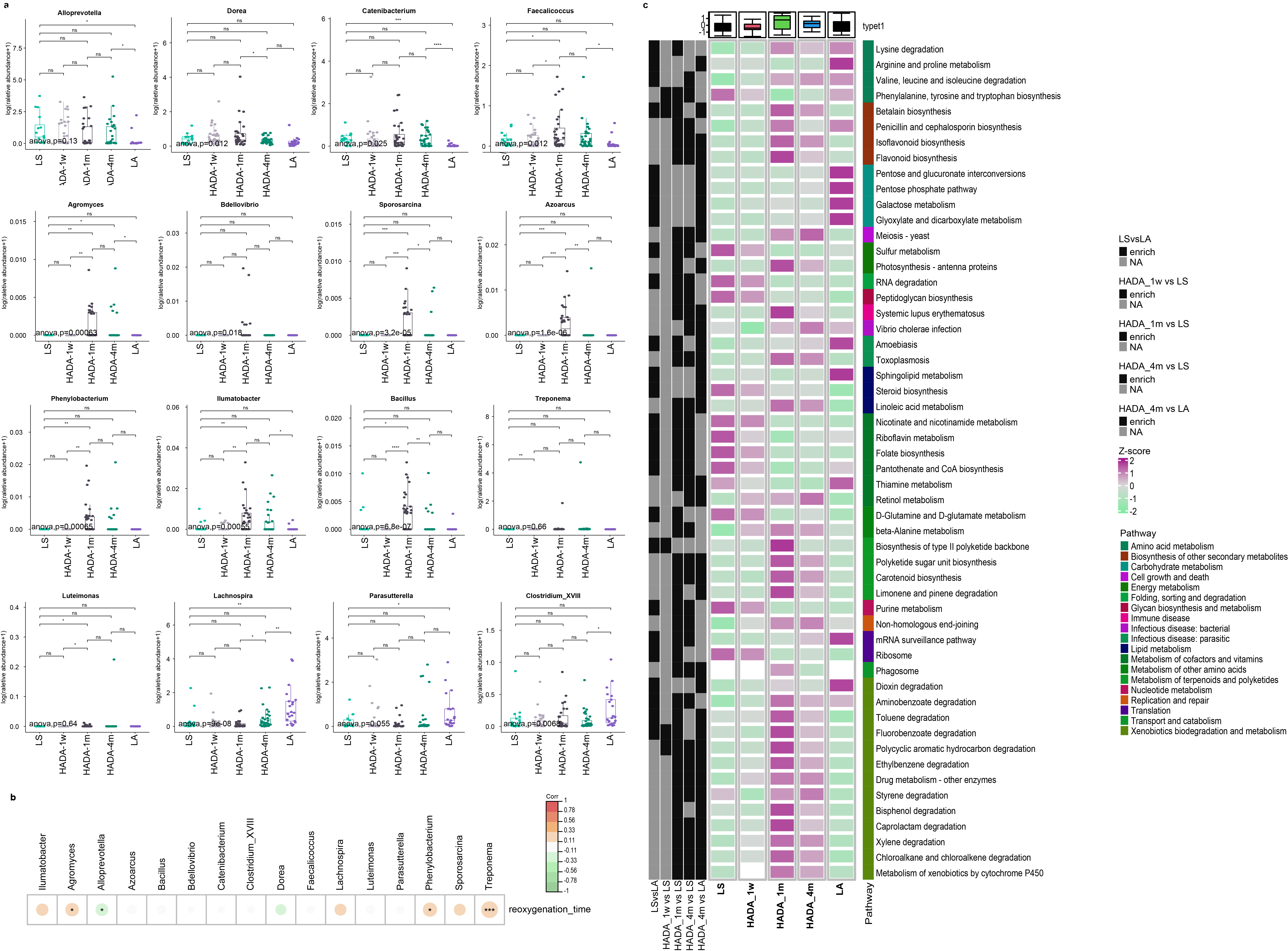


FigureS3. Verification of differences in key bacterial genera in LEfSe and prediction of intestinal bacterial community functions. a. Differential abundance of key bacterial genera in LEfSe in each group. b.Spearman correlation analysis between key bacterial genera and reoxygenation time of LEfSe. c. 16SrRNA KEGG pathway data predicted by PICRUSt. Only KEGG pathways predicted in at least two comparison groups are shown in the figure.


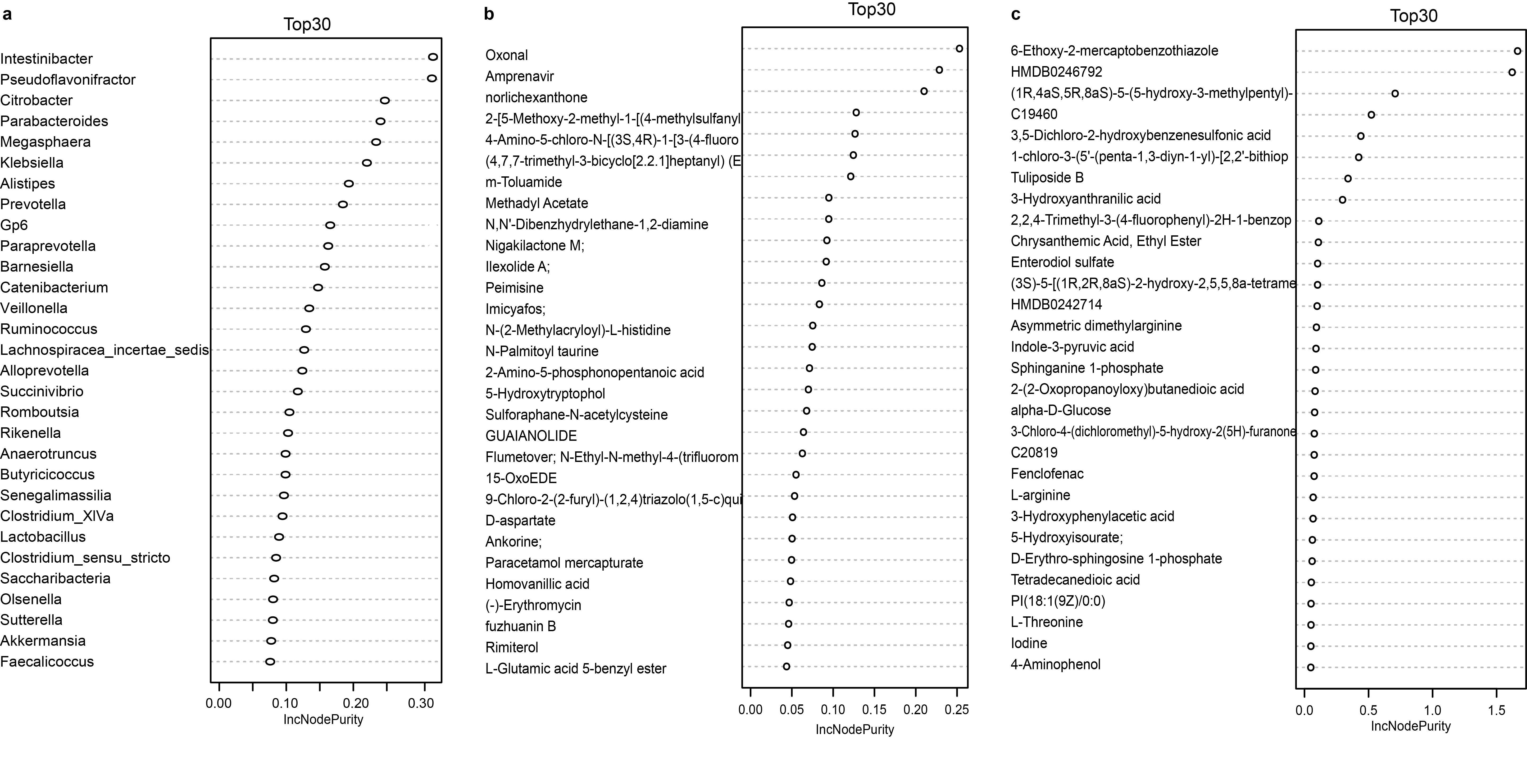


Figure S4. Random forest analysis for screening markers in multi-omics data of HADA_1w vs. LS group. a. Random forest screening of candidate reoxygenation-related biomarkers in gut microbiota dataset. b. Random forest screening of candidate reoxygenation-related biomarkers in fecal metabolome dataset. c. Random forest screening of candidate reoxygenation-related biomarkers in plasma metabolome dataset.


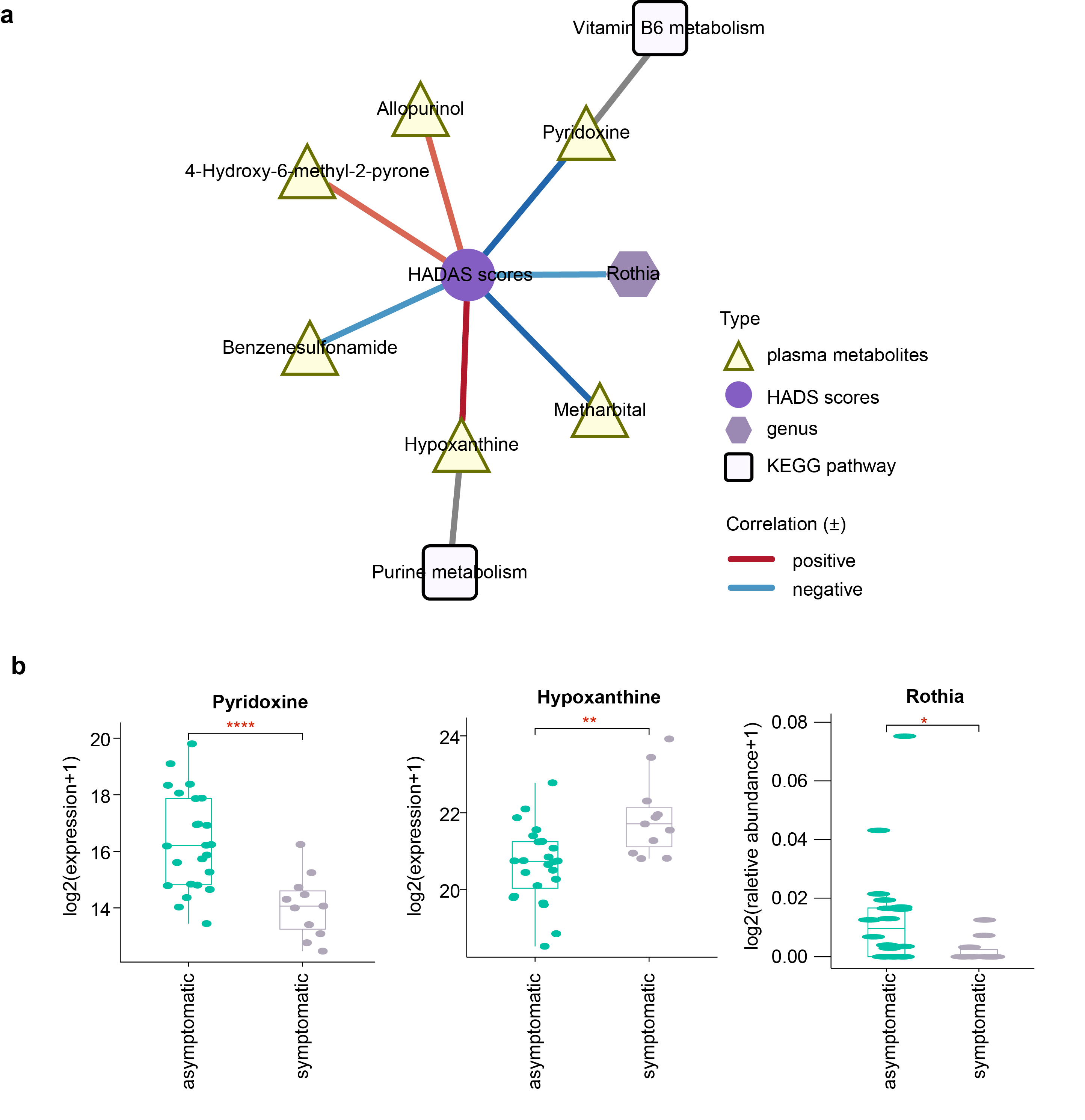


Figure S5. Correlation networks and differential metabolite-microbiome profiles during early reoxygenation in high-altitude residents. a. Spearman correlation network analysis between HADAS Scores, bacterial genera, and plasma metabolites during the first week of reoxygenation (HADA_1w) in high-altitude residents. Only correlation pairs with │r│>0.5 and p<0.05 are displayed in the figure. b. Boxplot plot illustrates the differential expression analysis of plasma metabolites (pyridoxine, hypoxanthine) and bacterial genus (*Rothia*) between the deacclimatization and asymptomatic groups within the HADA_1w group. A T-test was used to analyze the significance of these differences. *P<0.05, **P<0.01, ****P<0.0001.





Figure S6. Venn diagram analysis of differentially enriched KEGG pathways in fecal and plasma metabolites of high-altitude residents during prolonged reoxygenation. a. Venn diagram analysis of significantly enriched KEGG pathways for differential fecal metabolites in high-altitude residents during prolonged reoxygenation (HADA_4m) compared to their baseline at high altitude (LS) and to low altitude residents (LA). b. Venn diagram analysis of significantly enriched KEGG pathways for differential plasma metabolites in high-altitude residents during prolonged reoxygenation (HADA_4m) compared to their baseline at LS and to LA. Green circles represent the comparison group of LS vs LA. Orange circles represent the comparison group of HADA_4m vs LS. Purple circles represent the comparison group of HADA_4m vs LA.
